# Identification of immune-associated biomarkers of diabetes nephropathy tubulointerstitial injury based on machine learning: a bioinformatics multi-chip integrated analysis

**DOI:** 10.1186/s13040-024-00369-x

**Published:** 2024-07-01

**Authors:** Lin Wang, Jiaming Su, Zhongjie Liu, Shaowei Ding, Yaotan Li, Baoluo Hou, Yuxin Hu, Zhaoxi Dong, Jingyi Tang, Hongfang Liu, Weijing Liu

**Affiliations:** 1 Key Laboratory of Chinese Internal Medicine of Ministry of Education and Beijing, Dongzhimen Hospital, Beijing University of Chinese Medicine, Beijing, China; 2 Renal Research Institution of Beijing University of Chinese Medicine, Dongzhimen Hospital, Affiliated to Beijing University of Chinese Medicine, Beijing, China; 3 Beijing University of Chinese Medicine, Beijing, China

**Keywords:** Diabetes nephropathy, Tubulointerstitial injury, Bioinformatics, Machine learning, Immune infiltration

## Abstract

**Background:**

Diabetic nephropathy (DN) is a major microvascular complication of diabetes and has become the leading cause of end-stage renal disease worldwide. A considerable number of DN patients have experienced irreversible end-stage renal disease progression due to the inability to diagnose the disease early. Therefore, reliable biomarkers that are helpful for early diagnosis and treatment are identified. The migration of immune cells to the kidney is considered to be a key step in the progression of DN-related vascular injury. Therefore, finding markers in this process may be more helpful for the early diagnosis and progression prediction of DN.

**Methods:**

The gene chip data were retrieved from the GEO database using the search term ' diabetic nephropathy ‘. The ' limma ' software package was used to identify differentially expressed genes (DEGs) between DN and control samples. Gene set enrichment analysis (GSEA) was performed on genes obtained from the molecular characteristic database (MSigDB. The R package ‘WGCNA’ was used to identify gene modules associated with tubulointerstitial injury in DN, and it was crossed with immune-related DEGs to identify target genes. Gene ontology (GO) enrichment analysis and Kyoto Encyclopedia of Genes and Genomes (KEGG) pathway analysis were performed on differentially expressed genes using the ‘ClusterProfiler’ software package in R. Three methods, least absolute shrinkage and selection operator (LASSO), support vector machine recursive feature elimination (SVM-RFE) and random forest (RF), were used to select immune-related biomarkers for diagnosis. We retrieved the tubulointerstitial dataset from the Nephroseq database to construct an external validation dataset. Unsupervised clustering analysis of the expression levels of immune-related biomarkers was performed using the ‘ConsensusClusterPlus ‘R software package. The urine of patients who visited Dongzhimen Hospital of Beijing University of Chinese Medicine from September 2021 to March 2023 was collected, and Elisa was used to detect the mRNA expression level of immune-related biomarkers in urine. Pearson correlation analysis was used to detect the effect of immune-related biomarker expression on renal function in DN patients.

**Results:**

Four microarray datasets from the GEO database are included in the analysis : GSE30122, GSE47185, GSE99340 and GSE104954. These datasets included 63 DN patients and 55 healthy controls. A total of 9415 genes were detected in the data set. We found 153 differentially expressed immune-related genes, of which 112 genes were up-regulated, 41 genes were down-regulated, and 119 overlapping genes were identified. GO analysis showed that they were involved in various biological processes including leukocyte-mediated immunity. KEGG analysis showed that these target genes were mainly involved in the formation of phagosomes in Staphylococcus aureus infection. Among these 119 overlapping genes, machine learning results identified AGR2, CCR2, CEBPD, CISH, CX3CR1, DEFB1 and FSTL1 as potential tubulointerstitial immune-related biomarkers. External validation suggested that the above markers showed diagnostic efficacy in distinguishing DN patients from healthy controls. Clinical studies have shown that the expression of AGR2, CX3CR1 and FSTL1 in urine samples of DN patients is negatively correlated with GFR, the expression of CX3CR1 and FSTL1 in urine samples of DN is positively correlated with serum creatinine, while the expression of DEFB1 in urine samples of DN is negatively correlated with serum creatinine. In addition, the expression of CX3CR1 in DN urine samples was positively correlated with proteinuria, while the expression of DEFB1 in DN urine samples was negatively correlated with proteinuria. Finally, according to the level of proteinuria, DN patients were divided into nephrotic proteinuria group (*n* = 24) and subrenal proteinuria group. There were significant differences in urinary AGR2, CCR2 and DEFB1 between the two groups by unpaired t test (*P* < 0.05).

**Conclusions:**

Our study provides new insights into the role of immune-related biomarkers in DN tubulointerstitial injury and provides potential targets for early diagnosis and treatment of DN patients. Seven different genes ( AGR2, CCR2, CEBPD, CISH, CX3CR1, DEFB1, FSTL1 ), as promising sensitive biomarkers, may affect the progression of DN by regulating immune inflammatory response. However, further comprehensive studies are needed to fully understand their exact molecular mechanisms and functional pathways in DN.

**Supplementary Information:**

The online version contains supplementary material available at 10.1186/s13040-024-00369-x.

## Introduction

Diabetic nephropathy (DN), being a prevalent microvascular complication of diabetes mellitus, has emerged as the leading cause of end-stage renal disease on a global scale. The prevalence rates of both diabetes and DN have witnessed an upward trend in the past ten years(1–2).Epidemiological studies predict that the global diabetic population will reach 700 million by 2045, with 40% of diabetes patients developing DN due to long-standing disease progression. DN accounts for one-third of the burden of disability-adjusted life-years associated with chronic kidney disease [3–5].This highlights the severe global public health challenge posed by DN, imposing significant health and economic burdens on society. No specific treatment is currently available for DN, and management strategies mainly focuses on slowing the progression of the underlying primary disease [6]. Primary strategies for prevention and treatment encompass regulating glucose levels as well as managing blood pressure effectively. Additionally, inhibiting the renin-angiotensin-aldosterone system along with employing sodium-glucose co-transporter 2 inhibitors can be beneficial. Lifestyle modifications like encouraging physical activity, adopting dietary adjustments, and maintaining a healthy weight are also recommended [7–9]. Nevertheless, despite these efforts being implemented in clinical practice there remains an unmet need among patients with DN who unfortunately experience irreversible progression towards end-stage renal disease. Consequently, it is imperative for nephrologists and endocrinologists to comprehend the underlying causes behind DN’s development as well as its advancement mechanisms while simultaneously identifying dependable biomarkers that facilitate early detection leading to prompt intervention.

DN occurs as a result of metabolic and hemodynamic imbalances linked to long-term diabetes. Clinically, it is characterized by an increase in glomerular filtration rate (GFR), progressive elevation in urinary albumin excretion rate, and a sustained decline in GFR [10–12]. Morphologically, DN is distinguished by initial thickening of the glomerular basement membrane, accumulation of mesangial matrix, and damage to podocytes and tubular cells. In later stages, there is diffuse/nodular glomerulosclerosis along with tubulointerstitial fibrosis accompanied by inflammation [10]. Conventionally, DN has been primarily attributed to glomerular lesions. However, with further research into the mechanisms of tubulointerstitial oxidative stress injury, cellular apoptosis and autophagy, abnormal angiogenesis, and excessive inflammatory response activation in DN, it has been suggested that tubulointerstitial injury and interstitial fibrosis play significant roles in the occurrence and development of DN [13]. It is proposed that tubulointerstitial damage may occur earlier than glomerular lesions and can act as a relatively independent factor in predicting DN progression [14].

High blood glucose is a well-established risk factor for diabetic DN. However, high blood glucose alone cannot explain all the observed changes in renal tissue. Some researchers have suggested that the development and progression of DN may be linked to advanced glycation end-products, activation of protein kinase C, and overexpression of various growth factors. At present, the most commonly used clinical diagnostic markers of DN are eGFR and proteinuria [15]. It is generally believed that these two markers can predict the progression of DN and have a strong predictive ability for all-cause mortality in DN patients [16]. However, about 30% of DN patients can be characterized by urinary protein negative, positive urine protein can also be found in pathological types such as membranous nephropathy and focal stage glomerulosclerosis [17]. Therefore, the researchers further proposed renal tubular injury markers including N-acetyl-β-D-glucosaminidase (NAG), Liver-Type Fatty Acid-Binding Protein (L-FABP), Kidney Injury Molecule 1 (KIM-1), α1-microglobulin, Cystatin C and Neutrophil Gelatinase-Associated Lipocalin (NGAL) [18–21]. However, these indicators can be seen in a variety of kidney diseases and lack the specificity of diagnosis and prediction of DN. Recent studies indicate that the inflammatory process and immune cells might also play a role in this pathogenesis [22]. Immune cells are crucial in causing vascular damage associated with DN, and their migration to the kidneys is a significant step in the advancement of DN [23]. Diabetic renal tissue has been observed to experience infiltration by macrophages [24], and it has been proposed that products originating from these macrophages could trigger additional inflammation within diabetic kidneys(25–26). Additionally, activated T lymphocytes are associated with DN [27]. One of the most prominent features of leukocytes in diabetic patients is the activation state of neutrophils in the blood [28]. In this context, many types of pro-inflammatory molecules have been shown to play an important role in the development of DN [29]. Studies have shown that compared with macrophages incubated with the glomerular basement membrane of normal non-diabetic mice, the levels of TNF-α and IL-1β produced by macrophages incubated with the glomerular basement membrane of diabetic mice were significantly increased [30]. Human studies have shown that serum and urine TNF-α levels are significantly elevated in patients with diabetic kidney disease [31]. New studies have shown that DUSP1, PRDX6 and S100A8 may be related to changes in the immune microenvironment of DN patients [32]. Other inflammatory markers that have been studied include IL-6, Intracellular Adhesion Molecule 1 (ICAM-1), serum e-selectin, Plasminogen Activator Inhibitor-1 (PAI-1), Vascular Cell Adhesion Protein-1 (VCAM-1) and C-Reactive Protein (CRP) (33–34). However, these indicators did not explore the progress of DN from the perspective of changes in the level of inflammation in the renal tubulointerstitium. Immune cells are involved in vascular injury under DN conditions, and their migration to the kidney is a key step in disease progression (35–36). Therefore, finding markers in this process may be more helpful for the early diagnosis and progression prediction of DN.

Bioinformatics methods are widely used in identifying biomarkers associated with diagnosis, prognosis, and outcome prediction [37].Given the constraints of small sample size and potential inaccuracies in analyzing single-chip data [38], our research utilized a bioinformatics strategy that involved integrating multiple chips. This comprehensive approach allowed us to identify specific differentially expressed genes (DEGs) linked to tubulointerstitial injury in patients with DN. We conducted an extensive search within the GEO database, focusing on gene sequencing chips related to renal tubulointerstitial tissue from DN patients. To streamline our findings, machine learning (ML) algorithms were implemented for selecting and screening key genes. Machine learning is the intersection of statistics and computer science, which enables computers to gain insights from a wide range of data sets, thus providing an interesting landscape for building statistical models and improving data interpretation. Machine learning has been widely used in medical diagnosis in recent years because of its ability to crack complex patterns and relationships in large datasets(39–40). It can also play a role in disease prediction and optimization of treatment strategies(41–42). As far as DN is concerned, its early diagnosis is very important, but because there are few patients in this period to do renal biopsy, early diagnosis is very difficult. Therefore, most scholars use ML to predict the risk of DN and screen out factors such as age, race, and anti-diabetic drugs [43]. Some scholars also use ML to analyze the biomarkers [44] related to DKD and diabetic retinopathy in diabetic population. Liu et al. also used ML for pathological diagnosis of DN to improve its accuracy [45]. In conclusion, researchers are trying to use ML to explore other methods that can diagnose DN and try to improve the accuracy of diagnosis.

In this study, we assessed the relevance of these genes to clinical features and evaluated risk factors, aiming to uncover the molecular mechanisms involved in the pathophysiological processes related to DN tubulointerstitial injury and explore potential biomarkers. We also investigated the specific enrichment pathways and immune infiltration mechanisms of the identified genes. These discoveries offer a theoretical framework and empirical foundation for the early detection and focused intervention of DN.

## Materials and methods

### Data sources and preprocess

The search term “Diabetic nephropathy” was utilized to retrieve gene chip data from the GEO database (http://www.ncbi.nlm.nih.gov/GEO). The inclusion criteria were as follows: (1) human mRNA expression data set; (2) all samples consisted of tubulointerstitial tissue; (3) a case-control design was employed with sample sizes exceeding 3 for each group. After filtration, four public datasets, namely GSE30122, GSE47185, GSE99340, and GSE104954, containing gene expression profiles of human DN samples, were selected. With one glomerular DN dataset GSE96804 serving as the validation data set. The corresponding matrix datasets and platform files were downloaded, and their details are provided in Table 1 and Supplementary Table 1.

**Table 1:** Dataset details

Datasets	Platforms	Species	Tissue	Citation(s)	DN patients	Healthy controls	Time
GSE30122	GPL571(Affymetrix Human Genome U133A 2.0 Array)	Homo sapiens	Tubulointerstitium	PMID: 21,752,957 PMID: 26,190,114	10	24	2011
GSE47185	GPL11670(Affymetrix Human Genome U133 Plus 2.0 Array)	Homo sapiens	Tubulointerstitium	PMID: 23,950,145	18	6	2013
GSE99340	GPL19109 (Affymetrix Human Genome U133 Plus 2.0 Array)	Homo sapiens	Tubulointerstitium	PMID: 29,242,313 PMID: 30,511,699	18	7	2017
GSE104954	GPL22945(Affymetrix Human Genome U133 Plus 2.0 Array)	Homo sapiens	Tubulointerstitium	PMID: 29,724,730	17	18	2018
GSE96804	GPL17586(Affymetrix Human Transcriptome Array 2.0)	Homo sapiens	Glomeruli	PMID: 29,242,313 PMID: 30,511,699	41	20	2018

### Identification and functional enrichment analysis of differentially expressed genes in tubulointerstitial tissue of patients with DN

The identification of differentially expressed genes (DEGs) between DN and control samples was conducted using the “limma” package. DEGs were determined based on the following criteria: absolute |LogFC|> 0.5 and adjusted p-value < 0.05. Visualization of these DEGs was performed using the “ggplot2” package, employing a volcano plot [46]. To explore the main functions and pathways associated with the DEGs, gene set enrichment analysis (GSEA) was carried out to identify significant functional terms between the DN and control groups. The reference gene sets utilized in this analysis were obtained from the Molecular Signatures Database (MSigDB), specifically “c5.go.v7.4.symbols.gmt” and “c2.cp.kegg.v7.4.symbols.gmt”. A gene set was considered significantly enriched if it exhibited a P-value < 0.05 and a false discovery rate < 0.025.

### Weighted gene co-expression network analysis (WGCNA)

WGCNA is a method in systems biology that is used to analyze patterns of gene correlation across different samples [47]. It allows for the identification of genes that show highly coordinated changes in expression and helps identify potential core genes based on their co-expression within the expression matrix and their association with phenotypes. In this study, we utilized the R package “WGCNA” to identify gene modules linked to DN tubulointerstitial injury. The determination of soft-thresholding power β, which determines module scale independence and average connectivity under different weight coefficients, was done by evaluating module scale independence and average connectivity. We then constructed an unsigned topological overlap matrix (TOM) to measure dissimilarity (1-TOM) between genes. Hierarchical clustering and dynamic tree-cutting were applied for module detection. To assign genes with similar expression profiles to gene modules, we performed average linkage hierarchical clustering on the gene dendrogram using a “TOM-based” dissimilarity measure with a minimum gene set size of 50.For modules associated with clinical attributes, we calculated module membership as a representation of the correlation between specific genes and module eigengenes, while gene significance represented the correlation between specific genes and clinical variables. Finally, we visualized the gene network of characteristic genes to further analyze information within the modules.

### Selection and analysis of immune-associated DEGs in DN tubulointerstitial injury

We collected 2929 immune-associated genes from InnateDB and Immport (https://www.innatedb.ca/; https://www.immport.org/home), which are comprehensive datasets that curate immune-associated genes from research articles (Supplementary Table 2). The immune-associated DEGs were identified using the R package “limma” based on the merged gene expression matrix, with criteria of |LogFC|>0.5and adjusted *p*-value < 0.05. The expression of immune-associated genes was visualized through volcano and heatmap plots using the R packages “ggplot2” and “pheatmap”. We identified a set of target genes by intersecting immune-associated DEGs with important modules obtained through WGCNA. To gain further insight into the biological functions of these selected genes, we conducted Gene Ontology (GO) enrichment analysis and Kyoto Encyclopedia of Genes and Genomes (KEGG) pathway analysis on differentially expressed genes using the “ClusterProfiler” package in R [48]. We utilized a significance threshold of *P* < 0.05 to identify biological processes, cellular components, molecular functions, and signaling pathways. The protein-protein interaction network was constructed by inputting the target genes into the STRING database (https://STRING-db.org/). The MCC method of Cytoscape 3.9 was used to calculate the scores and build the protein-protein interaction network.

### Diagnostic immune-associated biomarker selection by machine learning algorithms

Machine learning algorithms are extensively utilized in the exploration of biomarkers due to their capacity to generate more refined models. In this particular investigation, three machine learning algorithms were employed: the Least Absolute Shrinkage and Selection Operator (LASSO), Support Vector Machine-Recursive Feature Elimination (SVM-RFE), and Random Forest (RF) [49]. The LASSO algorithm, implemented through the “glmnet” R package, conducts variable selection and complexity adjustment while fitting a generalized linear model [50]. SVM-RFE is a supervised machine learning technique that recursively ranks features based on their significance [51]. RF, on the other hand, iteratively evaluates and scores classification variables by constructing decision tree classifier models, resulting in highly accurate classification features [52]. These algorithms were collectively utilized to select core genes from the integrated chip dataset in this study.The genes identified from the overlap of the three analyses were considered as potential biomarkers associated with immune response in DN. Subsequently, by integrating the expression matrix of these biomarkers into the merged gene expression matrix, we observed their expression levels and generated co-expression pattern heatmaps using the “corrplot” package to investigate the relationships between their expressions.

### External validation of immune-associated biomarkers

The Nephroseq database (https://www.Nephroseq.org) is an online database commonly used for collecting gene expression data and clinical characteristics of kidney disorders and healthy control groups [53]. We retrieved four tubulointerstitial datasets from the Nephroseq database, namely ERCB Nephrotic Syndrome TubInt, Ju CKD TubInt, Schmid Diabetes TubInt, and Woroniecka Diabetes TubInt. We then constructed an external validation dataset comprising 46 patients with DN and 59 healthy controls. To assess the diagnostic efficacy of the biomarkers, receiver operating characteristic (ROC) curves were constructed using the merged gene expression matrix and the validation dataset from the Nephroseq database. A more comprehensive validation of the differentially expressed biomarkers was then performed based on the Nephroseq database, as well as a glomeruli dataset (GSE142025) obtained from the GEO database. Unpaired t-test was used with a significance threshold of *P* < 0.05 to determine whether there were differences in the expression of the selected biomarkers between the two groups.

### Gene set variation analysis (GSVA)

GSVA is an unsupervised and non-parametric method for gene set enrichment analysis, which evaluates the connection between biological pathways and gene signatures based on expression profiling [54]. A reference set of fifty hallmark gene sets was selected from MSigDB, representing well-defined biological states or processes with coherent expression patterns. The GSVA score for each gene set was calculated using the “ssGSEA” function in the “GSVA” package, indicating the level of absolute enrichment. To compare differences in GSVA scores between DN patients and healthy controls, we utilized the “Limma” package. Additionally, we employed the “reshape2” and “tidyverse” packages to determine correlations between immune-associated biomarkers and the 50 hallmark gene sets.

### CIBERSORT immune cell infiltration analysis

The CIBERSORT algorithm was employed to compute the ratios of different immune cells in the DN and control groups based on the merged gene expression matrix, and to visually represent the abundance of immune cells. A heat map illustrating the quantitative correlation between distinct immune cells was generated using the “corrplot” package. The proportions of 22 immune cells in DN and control groups were visualized using the R package “vioplot”, with a statistically significant difference (*P* < 0.05) indicating divergence between the two groups. Furthermore, we utilized the “ggplot2” R package to explore how expression levels of immune-associated biomarkers relate to variations in immune cell ratios.

### Consensus clustering analysis

We utilized the “ConsensusClusterPlus” R package to perform unsupervised clustering analysis on the expression levels of immune-associated biomarkers. The k-means algorithm was employed with 1,000 iterations for this analysis. By selecting a maximum subtype number of k (k = 9), we determined the optimal subtype number based on a comprehensive evaluation using the cumulative distribution function (CDF) curve, consensus matrix, and cluster score (> 0.9). To visually demonstrate the distribution difference between immune subtypes, we conducted PCA analysis and presented it graphically. To further elucidate the molecular characteristics of different subtypes, we extensively examined the expression differences of immune-associated biomarkers among the groups exhibiting different immune molecular patterns. Additionally, we compared the variations in immune infiltration and GSVA expression profiling between different subtypes.

### Collection of urinary samples and measurements of urinary mRNA levels

In this study, a cross-sectional survey was conducted to include 44 healthy controls, 56 patients who met the diagnostic criteria with type 2 diabetes mellitus (T2DM), and 45 patients with stage III-IV DN, who attended Dongzhimen Hospital of Beijing University of Traditional Chinese Medicine between September 2021 and March 2023. This clinical study received approval from the Medical Ethics Committee of Dongzhimen Hospital, affiliated with Beijing University of Chinese Medicine (in accordance with the 2021 Declaration of Helsinki, Ethical Batch No: 2022DZMEC-146). All participants were provided with a comprehensive explanation of the potential risks and benefits involved, and their informed consent was obtained. The gene names were initially converted to entrez IDs using the R package “org.hs.eg.db”. Subsequently, the four tubulointerstitial datasets were integrated using R (v4.2.0) to create a merged gene expression matrix. The R package “SVA,” which includes the “Combat” function [55], was employed to correct for batch effects among the different datasets. Principal component analysis (PCA) was then conducted to assess whether the batch effects had been eliminated (see Table 2).

**Table 2:** Distribution of general information of subjects [χ ± s/M(Q3-Q1)]

		Healthy control group (*n* = 44)	T2DM group (*n* = 56)	DKD group (*n* = 45)	χ^2^ / F / t	*P*
Gender, n(%)	male	24(55.5%)	30(53.6%)	21(46.7%)	0.678	0.713
	female	20(44.5%)	26(46.4%)	24(53.3%)		
Age (years)	-	52 ± 13	54 ± 14	61 ± 14	4.994	0.892
BMI(kg/m^2^)	-	24.85 ± 2.95	26.34 ± 3.68	26.28 ± 4.12	1.107	0.579
SBP(mmHg)	-	122 ± 10	134 ± 15	139 ± 21	6.378	0.014^*^
DBP(mmHg)	-	74 ± 8	81 ± 11	77 ± 15	2.437	0.108
T2DM duration of disease (years)	-	-	8 ± 7	16 ± 8	4.011	0.000^***^

Urine sample collection, processing, and storage were carried out in aseptic conditions by an experienced technician to prevent cross-contamination. Mid-stream urine samples were obtained during the medical checkup visits and subsequently centrifuged at 2,000 g for 20 min at room temperature. After centrifugation, the samples were stored at -80 °C until they were needed for further analysis. These procedures were conducted immediately after urine sample collection, Repeated freezing and thawing should be avoided, and the samples should be frozen within 6 months. After the samples were collected, Elisa was used to detect the mRNA expression levels of immune-associated biomarkers in urine. Finally, pearson correlation analysis was conducted to examine the influence of immune-associated biomarker expression on renal function in patients with DN, such as GFR, serum creatinine and proteinuria. The significance threshold was set at *P* < 0.05.

### Statistical analysis

All data calculations and statistical analyses were conducted using R software (version 4.2) or Origin 9.1. The diagnostic model’s predictive performance was assessed using ROC curves and determining the corresponding area under the curve (AUC) values. Pearson’s correlation analysis was employed to examine the relationships between variables. Furthermore, an unpaired t-test was utilized to analyze the differential expression levels of characteristic genes in DN. All statistical p-values were two-sided, and a *P* < 0.05 was considered statistically significant.

## Results

### Identification of DEGs in DN tubulointerstitial tissue

**Fig. 1:**
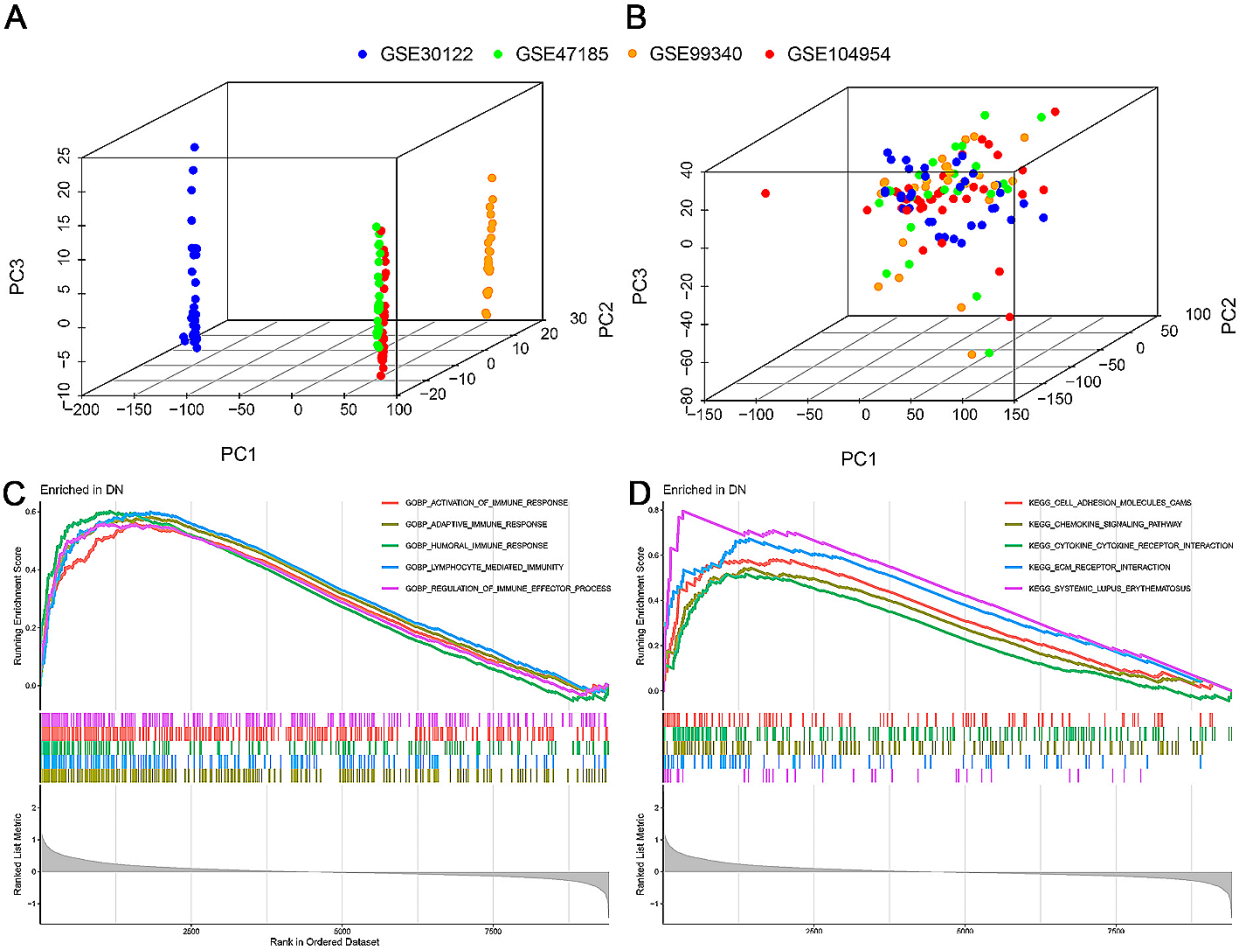
Identification of differentially expressed genes in the tubulointerstitial tissue of patients with DN. (**A**) Gene expression profiles without batch effect removal. (**B**) Gene expression profiles with batch effect removal. (**C**) Enriched active Gene Ontology functions in the DN tubulointerstitial gene expression matrix. (**D**) Enriched active KEGG pathways in the DN tubulointerstitial gene expression matrix

Four microarray datasets from the GEO database were included in the analysis: GSE30122, GSE47185, GSE99340, and GSE104954. These datasets included a total of 63 patients with DN and 55 healthy controls. After batch correction, the data distributions among the datasets became consistent, indicating successful normalization (Fig. 1A and B). A total of 9,415 genes were detected in the datasets. For the DN tubulointerstitial tissue, 454 DEGs were identified, including 272 upregulated genes and 182 downregulated genes (Supplementary Table 3). GSEA results on the 454 DEGs revealed that in the gene expression matrix of DN tubulointerstitial tissue, the enriched active GO functions mainly involved the activation of immune response, adaptive immune response, humoral immune response, lymphocyte-mediated immunity, and regulation of immune effector process (Fig. 1C). These findings strongly suggest the essential role of the immune response in DN. Furthermore, the enriched active KEGG pathways included extracellular matrix (ECM)-receptor interaction, cell adhesion molecules, chemokine signaling pathway, and cytokine-cytokine receptor interaction, among others (Fig. 1D).

### Weighted gene co-expression network construction

**Fig. 2:**
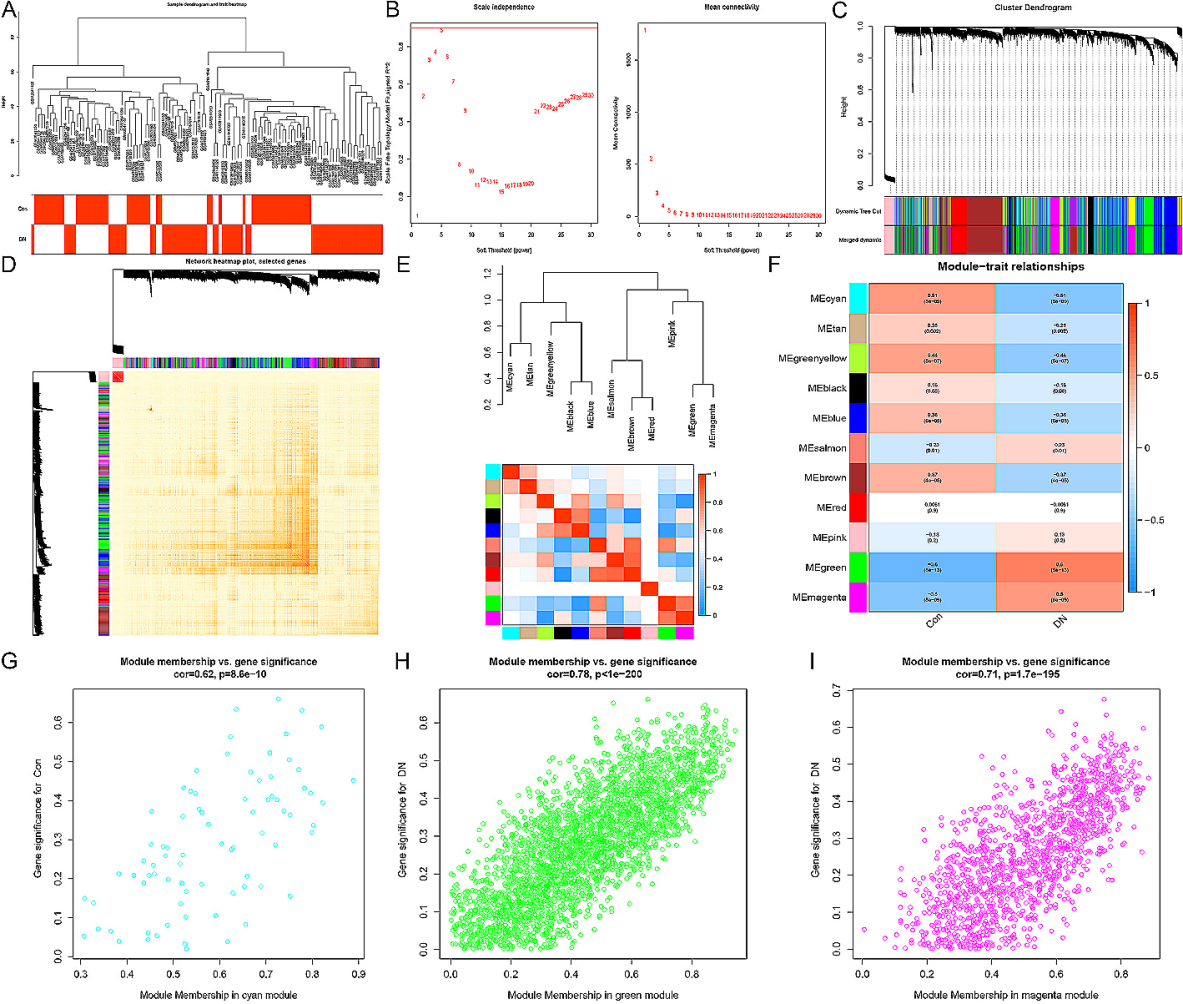
Construction of WGCNA co-expression network. (**A**) Sample clustering dendrogram with tree leaves corresponding to individual samples. (**B**) Selection of the optimal soft threshold. A value of five was determined as the best soft threshold. (**C**) Merging of similar modules. (**D**) Co-expression analysis of DEGs within each module.(**E**) Collinear heatmap of module feature genes. Red color indicates a high correlation, while blue color indicates opposite results.(**F**) Correlations between different modules and clinical traits. Red represents a positive correlation, and blue represents a negative correlation. (**G**-**I**) Significance of genes related to tubulointerstitial tissue in the cyan, green, and magenta modules. Each dot represents a gene within the respective module

This study utilized a WGCNA analysis on the merged gene expression matrix. A total of 63 DN samples and 55 healthy control samples were included for sample clustering, with any obvious abnormal samples being excluded based on a threshold (Fig. 2A). The soft threshold was set to 5 when R2 > 0.9 and the average connectivity was high, as depicted in Fig. 2B. By merging strongly associated modules using a clustering height limit of 0.25, we identified and visualized 11 modules in the clustering tree for further investigation (Fig. 2C). To confirm the reliability of module delineation, transcription correlation analysis revealed no significant correlations between modules (Fig. 2D). Additionally, there were no significant associations observed between modules when examining their correlations (Fig. 2E). We assessed the correlation between ME values (module eigengenes) and clinical features to explore the relationship between modules and clinical symptoms. Notably, we found that the cyan module exhibited a positive correlation with normal conditions (*r* = 0.51, *p* = 5e-09) but a negative correlation with DN cases (*r* = -0.51, *p* = 5e-09), while the green module showed an inverse pattern by displaying a negative correlation with normal conditions(*r*= -0.6,*p* = 5e-13)and positive association with DN cases(*r* = 0.6,*p* = 5e-13). Similarly, the magenta module displayed an opposite trend by showing negative association with normal conditions(*r*=-0.5,*p* = 8e-09)and positive association with DN cases(*r* = 0.5,*p* = 8e-09)(Figure F). Clinically relevant modules were identified, and it was found that both cyan, greeen, and magenta moduels had strong associationswithDN(Figure G-I). Subsequently, every gene within these three moduels underwent further examination.

### Identification of DEGs between immune-associated genes and critical module genes and functional analyses

**Fig. 3:**
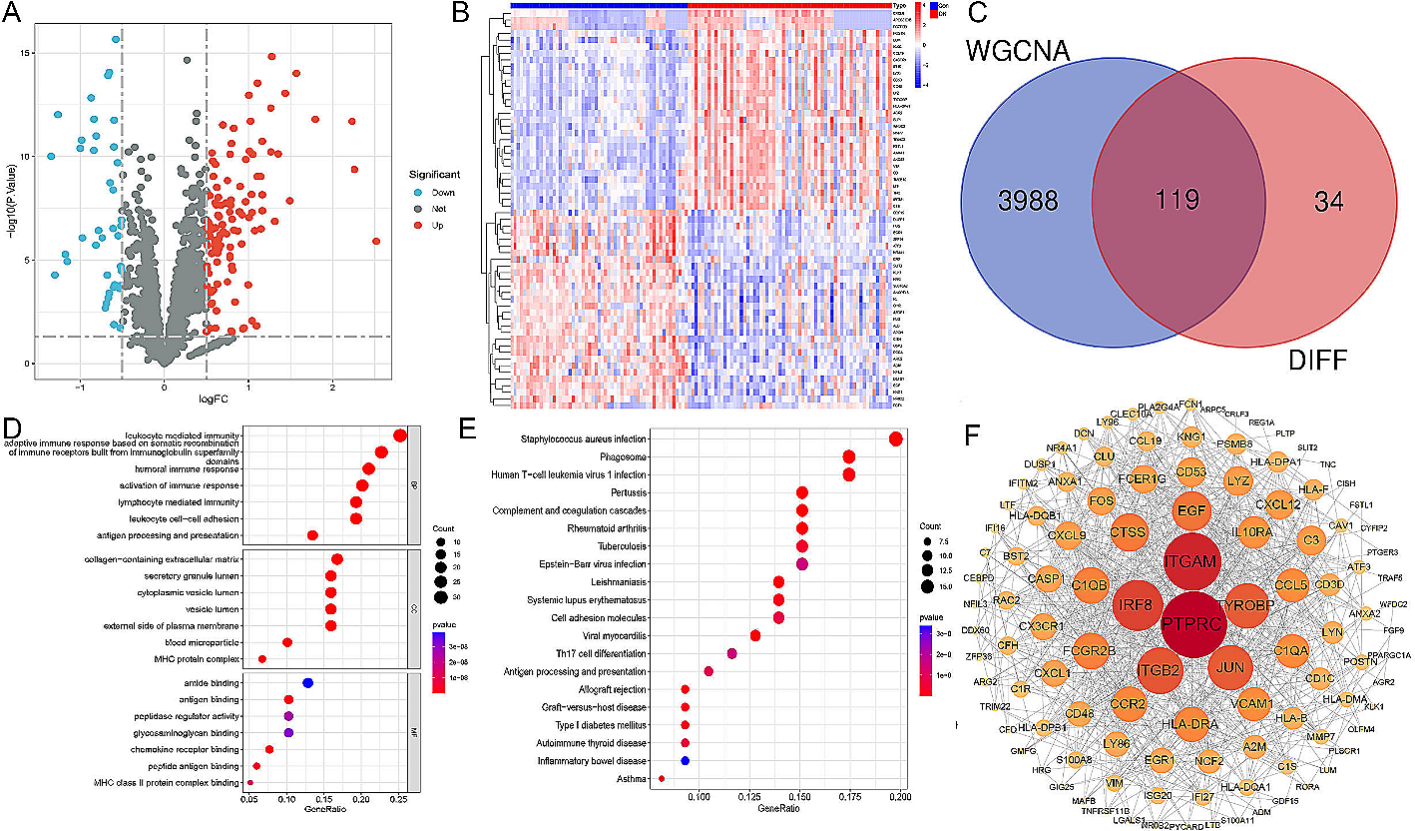
Identification of immune-associated DEGs in the modules based on the co-expression profile. (**A**-**B**) Volcano plots and heatmap depicting the differentially expressed immune-associated genes between the aforementioned groups. (**C**) Venn diagram showing the overlap between differentially expressed immune-associated genes and critical module genes. (**D**) GO analysis. (**E**) KEGG analysis. (**F**) The protein-protein interaction network of immune-associated DEGs in the modules generated by the STRING platform

After acquiring the genes associated with immunity, we compared their expression levels in healthy control samples and DN samples. We discovered 153 differentially expressed immune-associated genes, with 112 genes showing upregulation and 41 genes showing downregulation (Fig. 3A-B). Subsequently, we conducted an analysis to identify overlapping genes between the immune-associated genes and critical module genes using a Venn diagram. This analysis revealed 119 overlapping genes (Fig. 3C; Supplementary Table 4), which could potentially serve as gene targets related to immune-related tubulointerstitial injury in DN. To explore the biological functions of these differentially expressed immune-associated genes within the modules, we performed functional analysis. The Gene Ontology (GO) analysis indicated their involvement in various biological processes such as leukocyte-mediated immunity, humoral immune response, activation of immune response, lymphocyte-mediated immunity, and leukocyte cell-cell adhesion. Additionally, they were found to be associated with cellular components including collagen-containing extracellular matrix and MHC protein complex. Furthermore, these target genes exhibited molecular functions such as antigen binding, chemokine receptor binding, peptide antigen binding, and MHC class II protein complex binding (Fig. 3D). Moreover, our KEGG analysis demonstrated that these target genes primarily participate in pathways related to Staphylococcus aureus infection phagosome formation complement and coagulation cascades cell adhesion molecules Th17 cell differentiation antigen processing and presentation allograft rejection type I diabetes mellitus (Fig. 3E). In order to illustrate the interactions among these target genes more clearly a corresponding protein-protein interaction network was constructed (Fig. 3F).

### Identification of immune-associated biomarker via machine learning algorithm

**Fig. 4:**
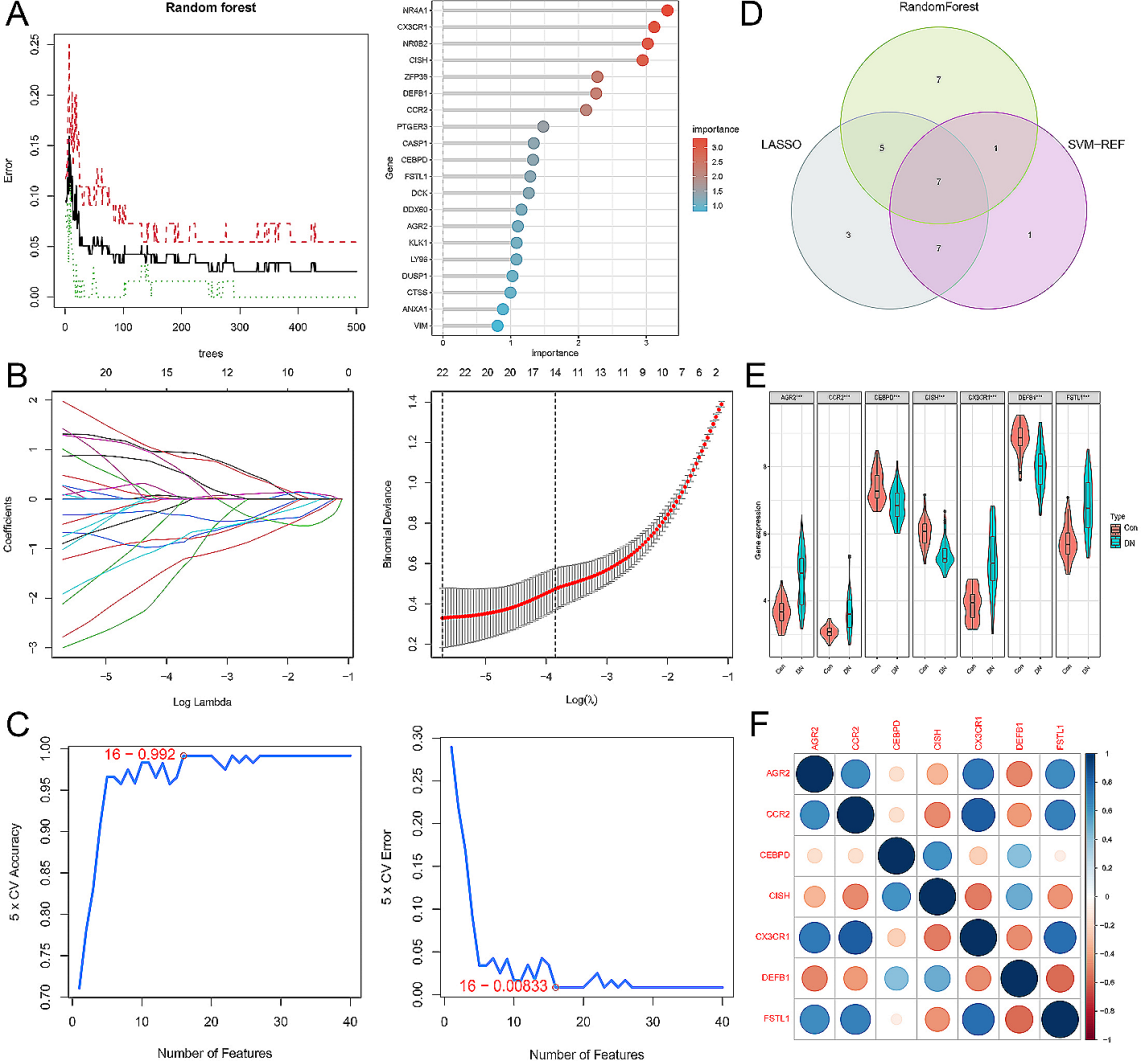
Selection of immune-associated biomarkers for DN with tubulointerstitial injury using machine learning algorithms. (**A**) Top 20 ranked feature genes from Random Forest analysis. (**B**) 22 feature genes obtained from LASSO analysis. (**C**) 16 feature genes identified by SVM-RFE analysis. (**D**) Venn diagram illustrating the overlap of selected feature genes. (**E**) Expression levels of biomarkers in the merged gene expression matrix. (**F**) Co-expression correlation heatmap showing the patterns of shared expression among the feature genes

Random Forest, LASSO, and SVM-RFE machine learning algorithms were utilized in this study to further investigate potential immune-associated biomarkers among the 119 key targets. The top 20 genes selected from the Random Forest analysis were considered as candidate biomarkers (Fig. 4A). A LASSO regression model was constructed, and cross-validation was performed, resulting in a minimum error corresponding to 22 candidate biomarkers (Fig. 4B; Supplementary Table 5). Additionally, the SVM-RFE analysis indicated that a model incorporating 16 genes achieved the highest accuracy and the lowest estimation error (Fig. 4C; Supplementary Table 6). By combining the results from the three algorithms, AGR2, CCR2, CEBPD, CISH, CX3CR1, DEFB1, and FSTL1 were identified as potential tubulointerstitial immune-associated biomarkers (Fig. 4D). We constructed seven biomarker expression patterns based on the merged gene expression matrix and observed distinct upregulation of AGR2, CCR2, CX3CR1, and FSTL1 expression in DN samples compared to healthy control samples, while CEBPD, CISH, and DEFB1 expression showed distinct downregulation in DN samples (Fig. 4E). Furthermore, the co-expression correlation heatmap displayed the interactions and relationships among these biomarkers (Fig. 4F).

### External validation of the immune-associated biomarkers

**Fig. 5:**
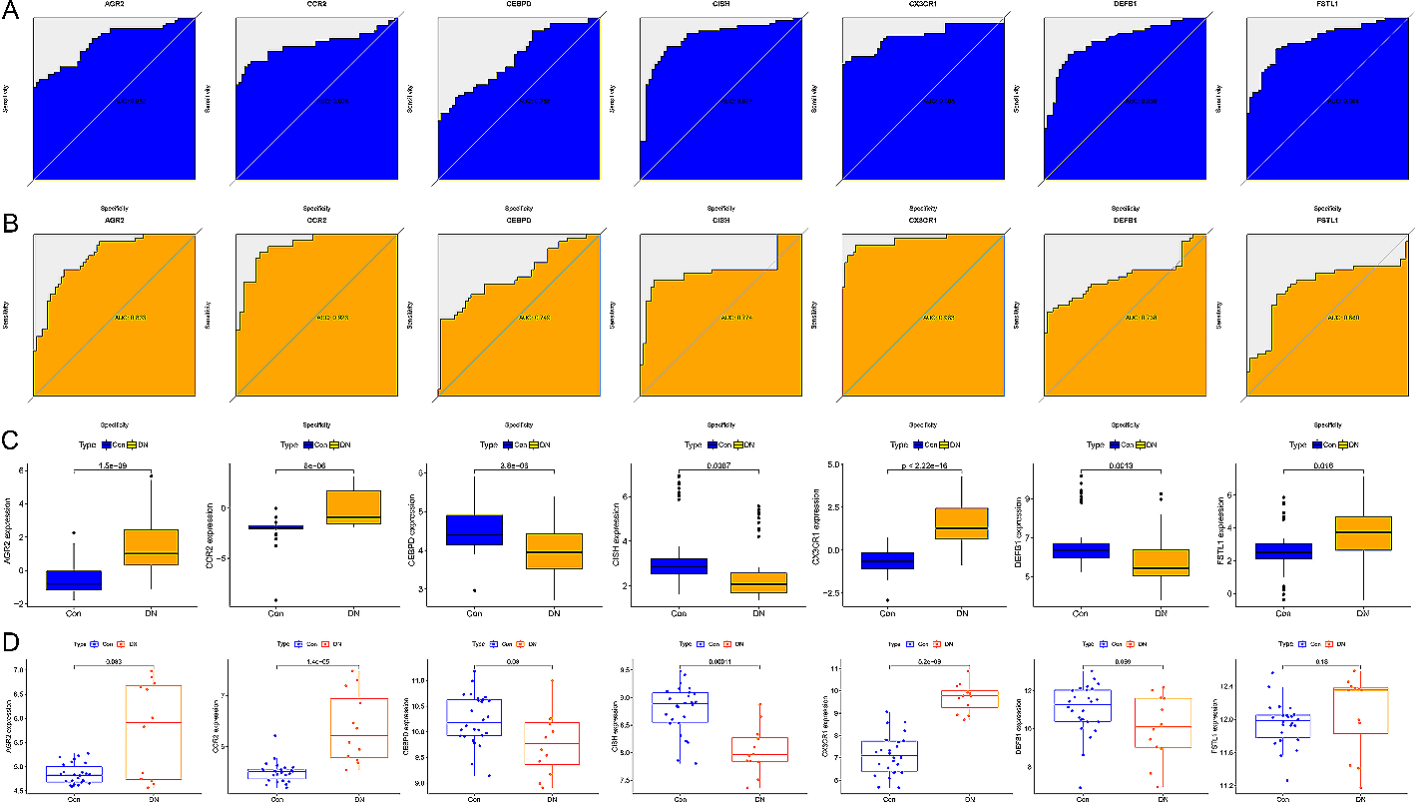
The verification results of immune-associated biomarkers. (**A**) ROC curves for each biomarker in the merged gene expression matrix to evaluate their diagnostic performance. (**B**) ROC curves for each biomarker in the Nephroseq v5 database to assess their diagnostic performance. (**C**) Representative boxplots illustrating the expression levels of characteristic genes in the Nephroseq v5 database. (**D**) Representative boxplots depicting the expression levels of characteristic genes in the glomeruli dataset (GSE142025)

We further confirmed the diagnostic potential of the immune-associated biomarkers by using ROC curves, and found that the 7 biomarkers identified in the merged gene expression matrix (AUC > 0.7) and the Nephroseq v5 database (AUC > 0.6) exhibited diagnostic efficacy in distinguishing patients with DN from healthy controls (Fig. 5A–B). Additionally, we validated the expression of these biomarkers in an external validation dataset constructed in Nephroseq v5 and the glomeruli (GSE96804) testing cohort, and consistent gene expression patterns were observed (Fig. 5C–D). Based on these findings, the above-mentioned genes demonstrated strong diagnostic power and potential to serve as diagnostic biomarkers for DN.

### The ssGSEA analysis of immune-associated biomarkers

**Fig. 6:**
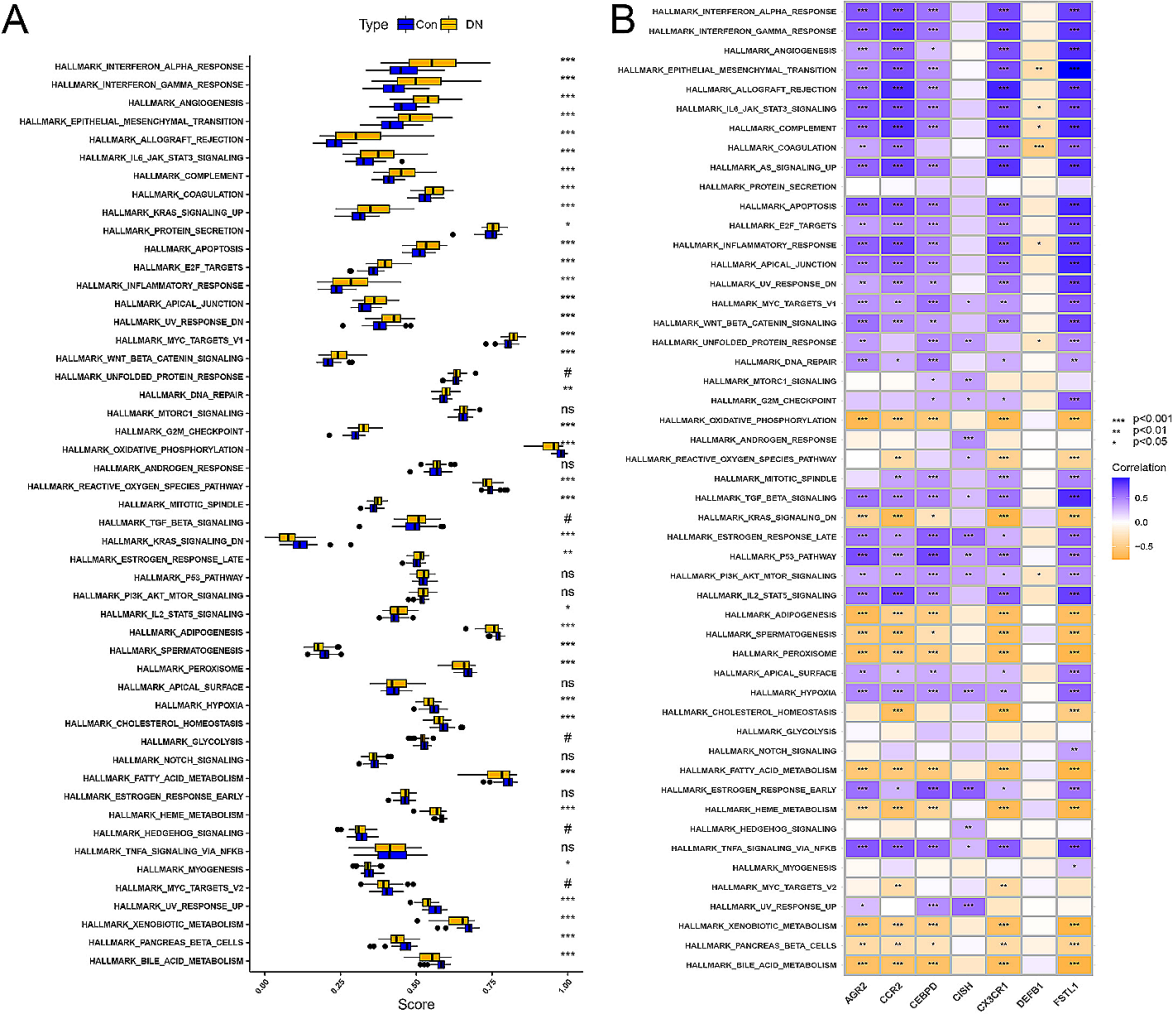
ssGSEA analysis of immune-associated biomarkers. (**A**) Differential activation of 50 hallmark gene sets. (**B**) Association between immune-associated biomarkers and the 50 hallmark gene sets

To refine and validate the biological characteristics of immune-associated biomarkers, enrichment analysis of the hallmark gene sets in the GSEA database was performed. The analysis revealed significant increases in several biological states or processes in patients with DN compared to healthy controls, including angiogenesis, epithelial-mesenchymal transition (EMT), IL6-JAK-STAT3 signaling, complement activation, apoptosis, and inflammatory response. Conversely, bile acid metabolism, pancreas β cells, and xenobiotic metabolism were significantly decreased in patients with DN (Fig. 6A). The majority of immune-associated biomarkers showed significant associations with these biological states or processes (Fig. 6B).

### Immune infiltration analysis

**Fig. 7:**
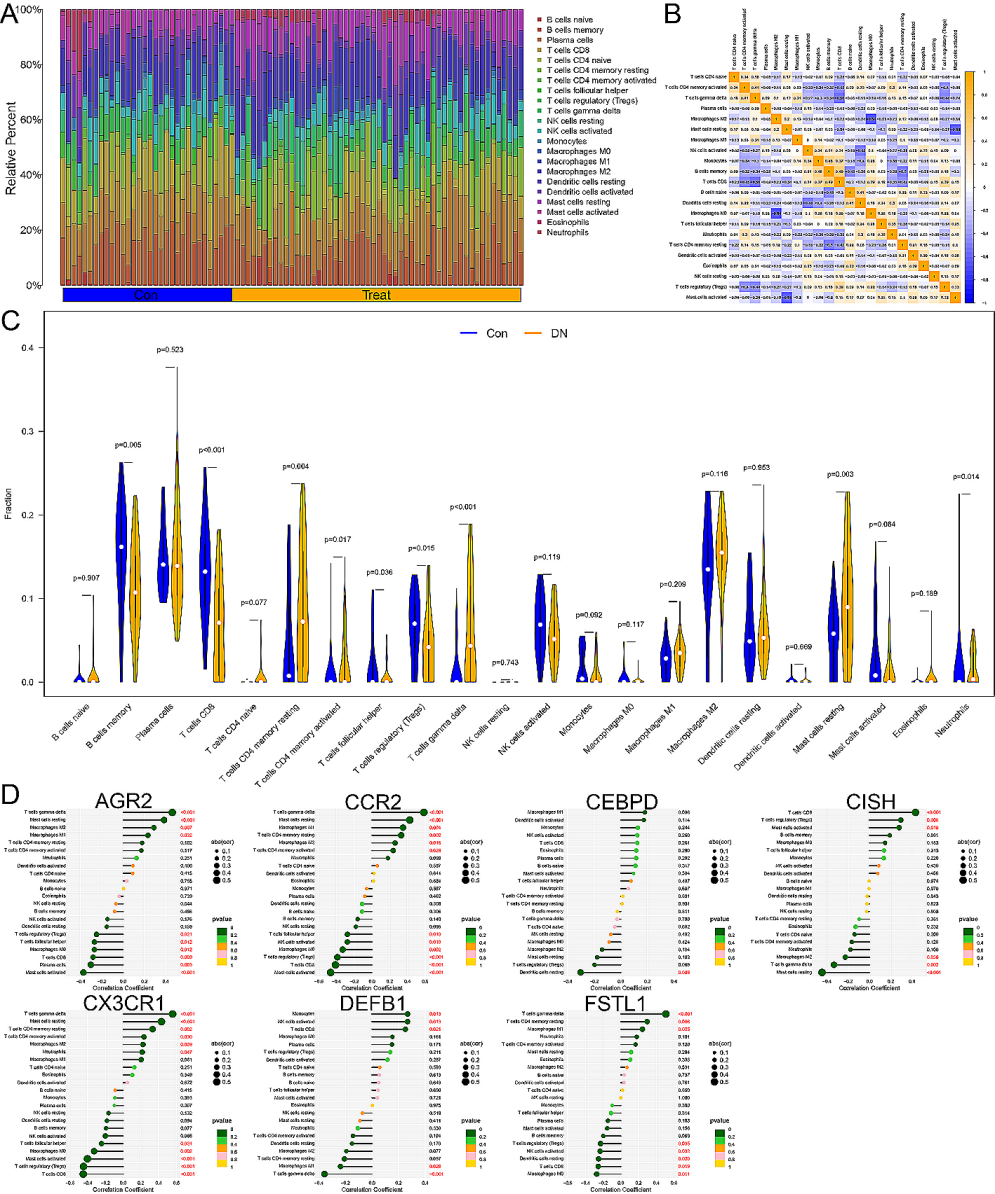
Infiltration of 22 types of immune cells. (**A**) Distribution of 22 types of immune cells in tissues of healthy controls and DN patients. (**B**) Heatmap of co-expression of the 22 types of immune cells. (**C**) The differences of infiltration of immune cells showed by violin diagram. (**D**) Visualization of Spearman correlation between immune cells and the immune-associated biomarkers

CIBERSORT algorithm was used to investigate the relative proportions of 22 types of immune cells in DN and control samples (Fig. 7A). Correlation analysis of all immune cells in the CIBERSORT algorithm revealed that macrophages M2 had the strongest negative correlation with macrophages M0 (*r* = -0.64), and T cells CD8 had the strongest positive correlation with B cells memory (*r* = 0.49) (Fig. 7B). The violin plot displayed that compared to the control group, the proportions of T cells CD4 memory resting (*P* = 0.004), T cells gamma delta (*P* < 0.001), Mast cells resting (*P* = 0.003), and neutrophils (*P* = 0.014) were significantly higher in DN samples, while the proportions of B cells memory (*P* = 0.005), T cells CD8 (*P* < 0.001), and T cells regulatory (*P* = 0.015) were lower (Fig. 7C). Via analyzing immune cell correlations, we determined that immune-associated biomarkers may play a role in the progression of DN by modulating immune cell populations such as T cells gamma delta, mast cells resting, mast cells activated, macrophages M1, macrophages M2, and T cells regulatory (Tregs) (Fig. 7D).

### Construction of two immune subtypes of DN tubulointerstitial injury based on immune-associated biomarkers

**Fig. 8:**
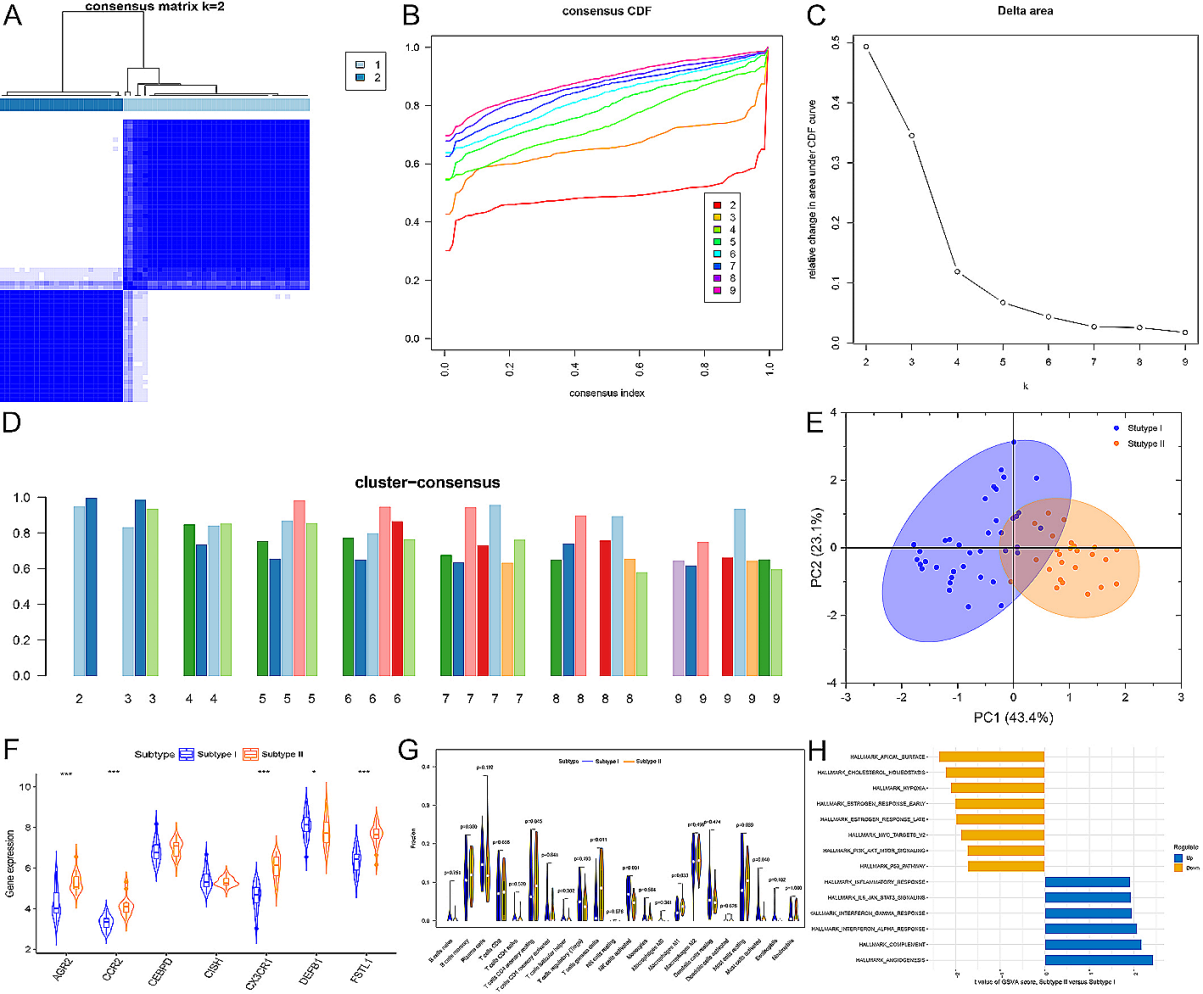
Identification of immune-associated molecular patterns in DN. (**A**) Consensus clustering matrix with k = 2. (**B**) Representative cumulative distribution function curves with k = 2 to 9.(**C**) Relative alterations in cumulative distribution function delta area curves. (**D**) Consensus score in each subtype with k = 2 to 9. (**E**) Principal component analysis analysis demonstrating the classification of patients with DN into two distinct subtypes. (**F**) Representative violin plots showing the expression of 7 characteristic genes between immune-associated subtypes. *p < 0.05. ***p < 0.001. (**G**) Representative violin plots showing the differences in infiltrated immune cells between immune subtypes. (**H**) Differences in the enriched hallmark gene sets between the two immune subtypes ranked by t-value of Gene Set Variation Analysis score

Utilizing the consensus clustering method, we grouped a total of 90 DN samples with tubulointerstitial injury based on the expression profiles of 7 immune-related biomarkers. Through analysis using a consensus matrix plot, a CDF plot, relative changes in the area under the CDF curve, and consistent cluster score (> 0.9) (Fig. 8A-D), we determined that there were two optimal subtypes. The distinction between these subtypes was evident in PCA analysis (Fig. 8E). We observed that subtype II exhibited significant upregulation of most immune-associated characteristic genes compared to subtype I (Fig. 8F). Furthermore, our findings revealed that subtype II had higher levels of infiltration by immune cell populations such as T cells CD4 memory resting, T cells gamma delta, macrophages M1, and mast cells resting (Fig. 8G). Additionally, pathway analysis demonstrated that subtype II displayed higher immune activation in pathways related to inflammation including complement activation, interferon response, IL6-JAK-STAT3 signaling pathway activation and inflammatory response when compared to subtype I (Fig. 8H). In conclusion, our study identified two distinct subtypes characterized by different immunological features and molecular mechanisms. Subtype II was classified as an immune subtype while subtype I was categorized as a non-immune subtype. These findings suggest that immune therapy may be beneficial for patients belonging to subtype II.

### Validation of urinary biomarker expression patterns

**Fig. 9:**
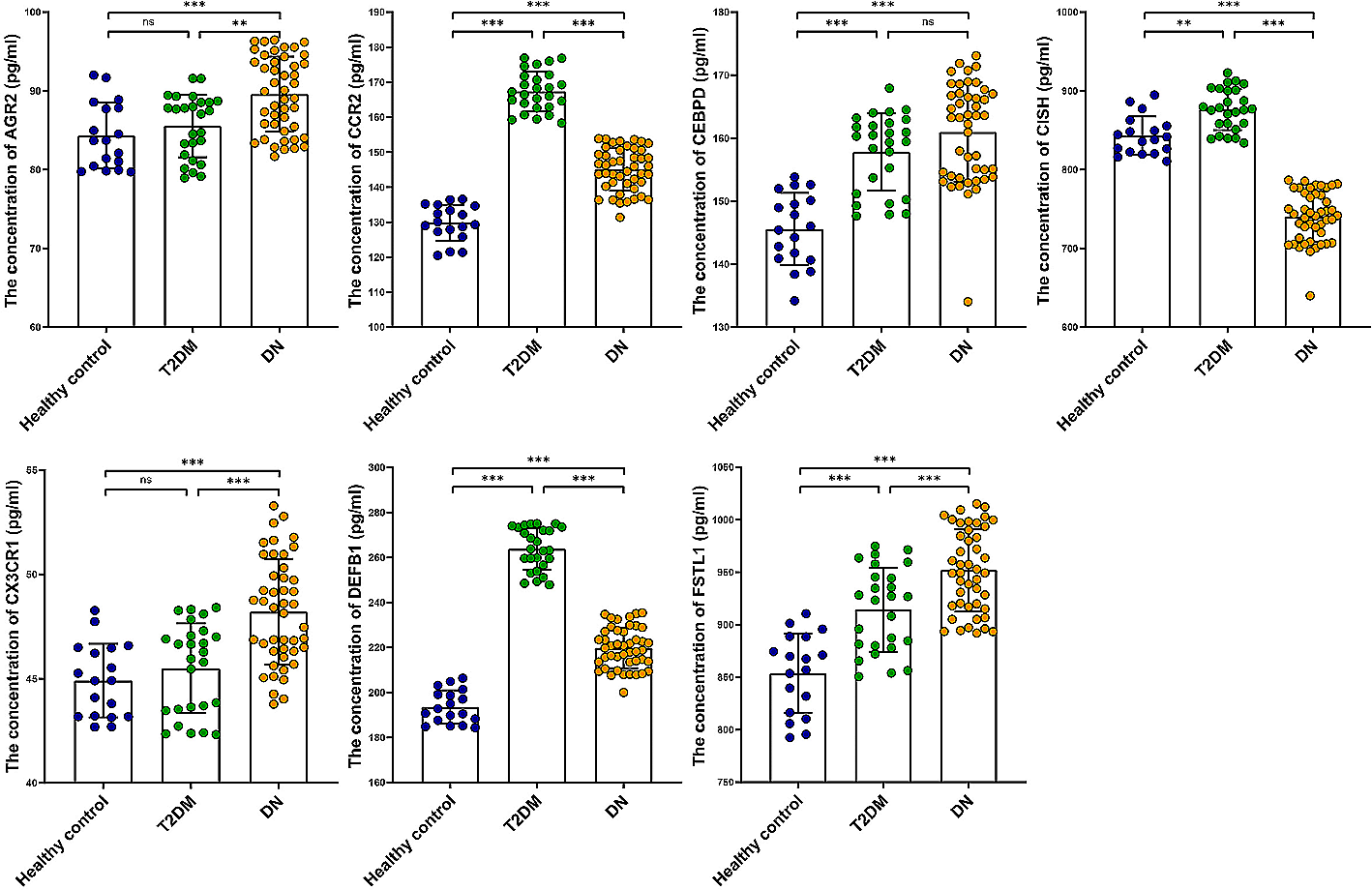
Validation of urine biomarker expression patterns. ns *P* > 0.05,^**^P < 0.01,^***^P < 0.001

We then measured the levels of each candidate characteristic gene in urine samples from healthy controls, patients with type II diabetes and patients with DN. Among the candidate characteristic genes, AGR2, CCR2, CEBPD, CX3CR1, DEFB1, and FSTL1 showed significantly higher expression in the urine of patients with DN compared to healthy controls (*P* < 0.05), while CISH showed significantly lower expression (*P* < 0.05) (Fig. 9). Among them, AGR2, CCR2, CISH, CX3CR1, and FSTL1were consistent with the results from GEO analysis, while the expression trend of CEBPD and DEFB1 was contrary to the prediction.

### Clinical correlation between immune-associated biomarkers and renal function in patients with DN

**Fig. 10:**
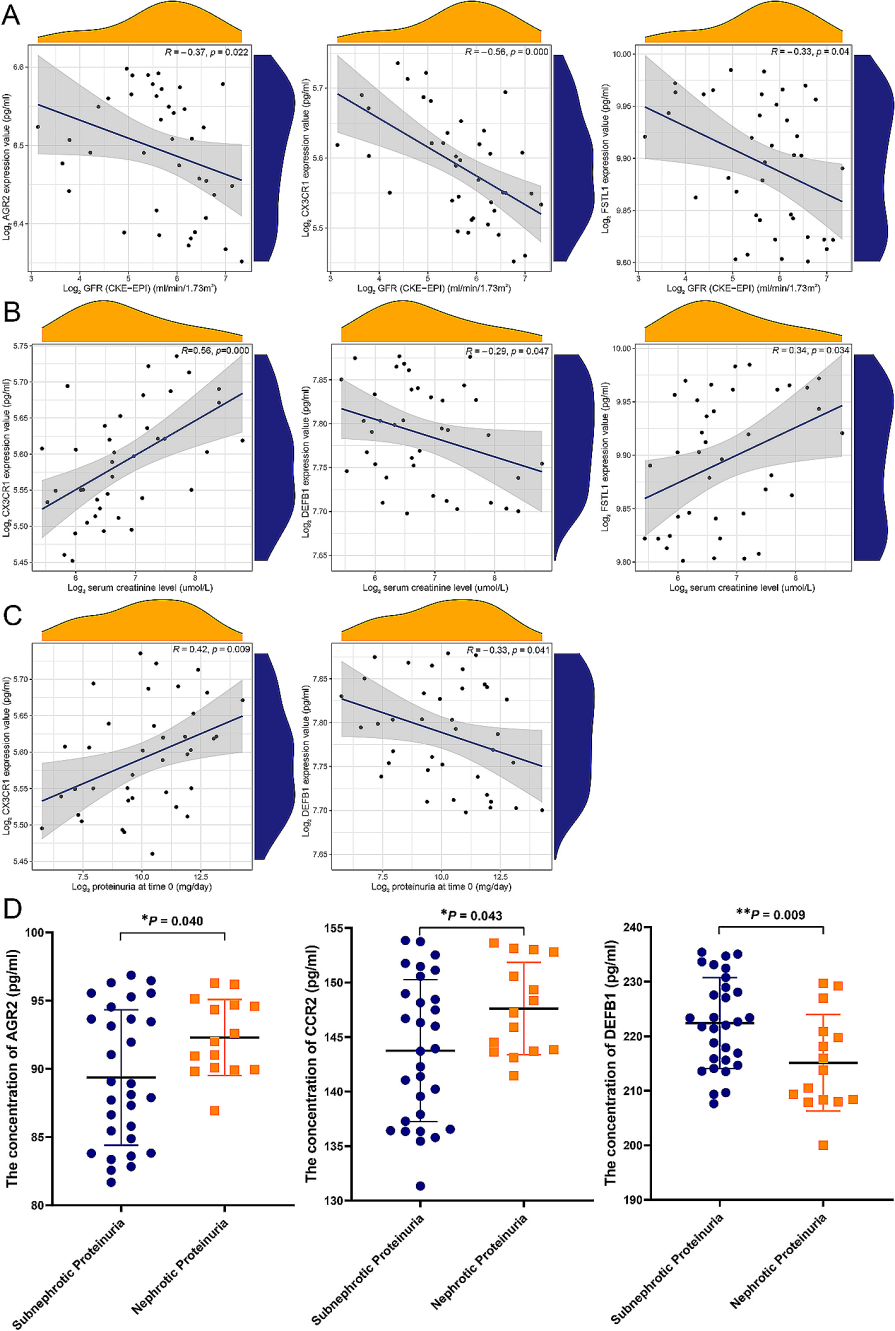
Clinical correlation analysis of core genes. (**A**) Correlation analysis between immune-associated biomarkers and glomerular filtration rate in patients with DN. (**B**) Correlation analysis between immune-associated biomarkers and serum creatinine in patients with DN. (**C**) Correlation analysis between immune-associated biomarkers and proteinuria in patients with DN. (**D**) Relationship between immune-associated biomarkers and proteinuria in patients with DN

To verify the potential roles of immune-associated biomarkers in DN progression, Pearson’s correlation analysis was conducted between the biomarkers and kidney function features in patients with DN. The results showed that the expression of AGR2, CX3CR1, and FSTL1 in DN urine sample was negatively correlated with GFR (Fig. 10A). Additionally, the expression of CX3CR1 and FSTL1 in DN urine sample was positively correlated with serum creatinine, while the expression of DEFB1 in DN urine sample was negatively correlated with serum creatinine (Fig. 10B). Moreover, the expression of CX3CR1 in DN urine sample was positively correlated with proteinuria, while the expression of DEFB1 in DN urine sample was negatively correlated with proteinuria (Fig. 10C). Finally, DN patients were divided into nephrotic proteinuria group (*n* = 24) and subnephrotic proteinuria group (*n* = 24) according to their proteinuria levels g/24 h). There were significant differences in urinary AGR2, CCR2, and DEFB1 between the two groups by unpaired t test (*P* < 0.05) (Fig. 10D). All suggesting the involvement of these immune-associated biomarkers in the development of DN. The rest of the correlations did not reach statistical significance and are not depicted in the figure.

## Discussion

DN is currently the most common chronic kidney disease and a leading cause of end-stage renal disease in adults, accounting for 40% of patients requiring renal replacement therapy. However, due to the limited accuracy of diagnostic biomarkers and the diverse nature of DN pathogenesis, the absence of pathological diagnosis in clinical practice has made it challenging to define and comprehend this condition. Consequently, numerous patients fail to achieve satisfactory outcomes [56]. Hence, there is an urgent requirement to identify more effective diagnostic biomarkers and appropriate molecular subtypes while establishing a diagnostic model for DN. Immune and inflammatory mechanisms play a significant role in both the development and progression of DN. Tubulointerstitial changes, including peritubular fibrosis, can contribute to or even propel the advancement of DN; however, these alterations are often disregarded. The tubulointerstitial injury observed in DN does not arise as a result of glomerular lesions but rather exists during early stages and significantly influences disease progression [57–58]. Therefore, investigating relevant biomarkers becomes crucial for early detection of DN. In this study, we utilized tubulointerstitial gene expression profiling data from patients with DN obtained from the GEO database until January 2022. By integrating bioinformatics methods and machine learning techniques, we aimed to explore the underlying molecular mechanisms and clinical diagnostic value.

In the current study, we developed a comprehensive and in-depth evaluation system to analyze immune-associated genes and signaling pathways involved in.First of all, bioinformatics multi-chip integrated analysis combined with WGCNA, a total of 119 potential gene targets associated with immune-related DN tubulointerstitial injury were identified between 90 DN patients and 100 healthy controls from the GEO database, which became the focal point of our research. Subsequent bioinformatics analysis revealed that the immune-associated DEGs were mainly enriched in various proteinase inhibitor activities, humoral immune response, cell adhesion, collagen-containing extracellular matrix-related GO functions, as well as signaling pathways such as Staphylococcus aureus infection, Phagosome, complement and coagulation cascades, cell adhesion molecules, and NF-kappa B signaling pathway according to KEGG analysis. Suggesting that these DEGs were closely related to the Renal tubulointerstitial immune verification reaction pathological changes of DN.

Machine learning algorithms are increasingly employed to develop decision models that assist in the identification and treatment of diseases. In this study, we validated seven potential biomarkers (AGR2, CCR2, CEBPD, CISH, CX3CR1, DEFB1, and FSTL1) associated with immune-related DN tubulointerstitial injury. The validation process involved integrating the results from three machine learning models and selecting additional verification sets. Moreover, We preliminarily validated the differential expression of seven biomarkers in urine of DN patients by a cross-sectional survey of clinical subjects, and their association with renal function indicators to verify their clinical value.

### Anterior gradient 2 (AGR2**)**

AGR2 belongs to the superfamily of protein disulfide isomerases, a group of enzymes primarily expressed in the endoplasmic reticulum (ER) [59]. AGR2 may disrupt the formation of disulfide bonds, cysteine-based redox reactions, and protein quality control processes within the ER. Its role in maintaining ER homeostasis (proteostasis) is crucial [60–62]. Previous research has demonstrated that AGR2 is highly expressed in various human cancers and plays a functional role in promoting the progression and metastasis of adenocarcinomas [63]. Zhou et al. identified AGR2 as one of the key genes involved in diabetic nephropathy glomerular lesions, independent of hypertensive nephropathy and three types of glomerulonephritis: membranous nephropathy, focal segmental glomerulosclerosis, and immunoglobulin A nephropathy [64]. Additionally, a recent study observed a significant increase in AGR2 expression in pancreatic tissue following treatment with Dapagliflozin using a diabetic mouse model. However, further investigation is needed to understand its contribution to tubular lesions development in diabetic nephropathy [65]. Given the crucial role of AGR2 in ER homeostasis, its increased expression is speculated to be mediated by ER stress and may serve as a marker and potential therapeutic target for tubulointerstitial damage in DN.

### Chemokine (C-C motif) receptor 2 (CCR2)

CCR2 is a receptor for chemokines that regulates immune responses by promoting the migration of macrophages and monocytes to the site of inflammation. This receptor has been found to play a role in glomerulosclerosis [65–67]. Preclinical studies have suggested that CCR2 antagonists may have potential benefits in improving insulin resistance by modulating adipose tissue, as well as restoring renal function through metabolic and anti-fibrotic effects in mice with type 2 diabetes(68–69). Kitagawa et al. were the first to demonstrate the preventive effects of CCR2 antagonists on progressive renal injury characterized by fibrosis associated with infiltration of inflammatory cells [70]. In humans, recent clinical trials have reported the renal protective effects of CCR2 inhibitors (CCR2 antagonism) in DN, reducing inflammatory responses and effectively reducing albuminuria and/or proteinuria. In summary, CCR2 is a promising diagnostic biomarker and therapeutic target [71].

### CCAAT/enhancer binding protein (CEBPD)

CEBPD belongs to the CCAAT/enhancer-binding protein family and is involved in regulating immune responses as well as cell growth, arrest, and differentiation. It is associated with various diseases characterized by altered inflammatory responses [72], such as cancer, atherosclerosis, and Alzheimer’s disease [73]. Current research indicates that CEBPD acts as a pro-inflammatory transcription factor, enhancing the expression of pro-inflammatory mediators. However, its mechanism of action in tubulointerstitial injury in DN remains largely unknown [74–76].

### Cytokine-inducible SH2-containing (CISH)

The cytokine signaling pathway plays a crucial role in various immune processes, and CISH, as a member of the suppressor of cytokine signaling family(77–78), acts as an important negative regulator of signal transduction and function in T cells and natural killer cells, plays a crucial role in various immune processes [79]. This study is the first to report the differential expression of CISH in renal tissue gene chip and urine in DN patients, and the association between CISH and renal tubulointerstitial damage in DN depends on further studies.

### Fractalkine receptor chemokine (C-X3-C motif) receptor 1 (CX3CR1)

In the development of kidney diseases, the chemotaxis and adhesion of immune cells are facilitated by CX3CR1 and its highly selective ligand CX3CL1 [80]. This leads to the infiltration of immune cells in the local area, resulting in the production and release of inflammatory and pro-fibrotic cytokines (such as TNF-α, IL-1β, and interferon-γ) as well as various growth factors. Consequently, this process contributes to the formation of extracellular matrix and subsequent damage to the kidneys (81–82). Hence, it is believed that CX3CR1 expression along with its ligand plays a crucial role in determining disease severity in kidney conditions like IgA nephritis, systemic lupus erythematosus, and inflammatory disorders such as transplant rejection(83–84). The effectiveness of immunotherapeutic inhibition targeting CX3CR1 has been demonstrated against glomerulonephritis associated with kidney diseases [85–87]. Moreover, studies indicate that regardless of the underlying disease type, there may be involvement of the CX3CL1/CX3CR1 axis in tubulointerstitial fibrosis within the kidneys. Elevated levels of CX3CR1 have been observed in renal tissues affected by fibrosis compared to those without fibrotic or non-inflammatory kidney diseases [88]. Additionally, increased mRNA expression for CX3CR1 has been detected during early stages of DN.CX3CR1 knock out can improve kidney damage in streptozotocin-induced diabetic mice, reducing ECM deposition and macrophage infiltration, thereby alleviating renal fibrosis and inflammation [89]. These findings collectively suggest that CX3CR1 could be an effective therapeutic target for preventing diabetic kidney disease [90]. However, there is limited clinical research on the expression of CX3CR1 in DN. Therefore, we investigated the expression of CX3CR1 in the urine of DN patients. It was confirmed that its differential expression has a certain correlation with renal injury related indicators.

### Human β-defensin-1 (DEFB1)

DEFB1 belongs to the defensins antimicrobial peptide family and is considered one of the most important antimicrobial peptides in the epithelial tissues of the urinary and respiratory tracts [91]. Oh et al., found that the serum levels of DEFB1 were higher in patients with DN compared to the healthy control group [92]. In contrast, DEFB1 was found to be down-regulated in renal tubulointerstitial tissues and up-regulated in urine in DN patients.

### Follistatin-like protein 1 (FSTL1)

FSTL1 is a fibroblast-derived cytokine that is closely associated with fibrosis in various organs such as the kidney, liver, and lungs((93–94). Clinical and basic research have confirmed that FSTL1 is upregulated in patients with chronic kidney disease and is involved in promoting renal fibrosis, inflammation, and cellular apoptosis processes.This is consistent with our results of urine from DN patients. However, FSTL1 remains to be further investigated as a promising clinical biomarker for renal tubulointerstitial injury in DN.The ssGSEA analysis indicate that the dysregulated expression of selective genes is associated with the pathological and physiological effects of DN. It is speculated that these biomarkers, through interplay with multiple signaling pathways, exacerbate epithelial-mesenchymal transition, metabolic dysregulation, and immune microinflammation, collectively promoting pathological changes in renal fibrosis and leading to progressive deterioration of renal function [95]. Although DN is not primarily an immune-mediated kidney disease, extensive evidence supports the involvement of both innate and adaptive immune pathways in the progression of renal dysfunction associated with DN(96–97). Therefore, understanding the implications of biomarkers during immune infiltration progression becomes crucial [95].

We observed significant variations in the composition and abundance of infiltrating immune cell populations between the two groups, highlighting the pivotal role of immune cells in the development of DN. In addition, we performed an unsupervised clustering approach to construct two subtypes based on the expression profiles of the seven biomarkers. Subtype II shows higher levels of immune cell infiltration and activation of inflammation-related pathways, and it is reasonable to infer that subtype 2 May be more closely related to immune-related tubulointerstitial injury in DN, which may be helpful for early diagnosis and intervention of DN.

The present study is subject to some limitations. Firstly, the study was based on public database, therefore further clinical and experimental research is needed to validate the detection methods and specific applications of biomarkers in terms of their scope and accuracy. Moreover, The clinical sample size was limited, and some clinicopathological features such as specific clinical classification, follow-up information and complications were not considered in our study, which may have some bias on the data, and further clinical investigation on a larger scale is needed. Lastly, the potential effects of biomarkers on the immune system warrant further investigation, and basic research needs to be further carried out in the future to consolidate the research conclusions.

## Conclusions

In summary, our study offers novel insights into the role of immune-related biomarkers in renal tubulointerstitial injury in DN and presents potential targets for early diagnosis and treatment of DN patients. Furthermore, we have initially identified seven distinct genes (AGR2, CCR2, CEBPD, CISH, CX3CR1, DEFB1, and FSTL1) as promising sensitive biomarkers that may influence the progression of DN through the regulation of immune inflammatory response. However, further comprehensive investigations are required to fully understand their precise molecular mechanisms and functional pathways in DN.

### Electronic supplementary material

Below is the link to the electronic supplementary material.

Supplementary Material 1

Supplementary Material 2

Supplementary Material 3

Supplementary Material 4

Supplementary Material 5

Supplementary Material 6

Supplementary Material 7

Supplementary Material 8

## Data Availability

No datasets were generated or analysed during the current study.

## Code availability

https://github.com/sujiaming0627/Identification-of-Immune-Associated-Biomarkers-of-Diabetes-Nephropathy-Tubulointerstitial-injury.

## Declarations

### Disclaimer

All claims expressed in this article are solely those of the authors and do not necessarily represent those of their affiliated organizations, or those of the publisher, the editors and the reviewers. Any product that may be evaluated in this article, or claim that may be made by its manufacturer, is not guaranteed or endorsed by the publisher.
