# Supplementary material for: Identification of immune-associated biomarkers of diabetes nephropathy tubulointerstitial injury based on machine learning: a bioinformatics multi-chip integrated analysis
Source: BioData Min. 2024 Jul 1;17:20. doi: 10.1186/s13040-024-00369-x (PMC11218417; doi:10.1186/s13040-024-00369-x)
Supplement: Supplementary file 2 — Supplementary Material 2 [file 13040_2024_369_MOESM2_ESM.docx]

**Supplementary TABLE 1:** The exact sample assignments of the multi-chip dataset and validation data set.

| Sample | set | condition |
| --- | --- | --- |
| GSM757024 | training | healthy |
| GSM757025 | training | healthy |
| GSM757026 | training | healthy |
| GSM757027 | training | healthy |
| GSM757028 | training | healthy |
| GSM757029 | training | healthy |
| GSM757030 | training | healthy |
| GSM757031 | training | healthy |
| GSM757032 | training | healthy |
| GSM757033 | training | healthy |
| GSM757034 | training | healthy |
| GSM757035 | training | healthy |
| GSM758498 | training | healthy |
| GSM758499 | training | healthy |
| GSM758500 | training | healthy |
| GSM758501 | training | healthy |
| GSM758502 | training | healthy |
| GSM758503 | training | healthy |
| GSM758504 | training | healthy |
| GSM758505 | training | healthy |
| GSM758506 | training | healthy |
| GSM758507 | training | healthy |
| GSM758508 | training | healthy |
| GSM758509 | training | healthy |
| GSM1146427 | training | healthy |
| GSM1146428 | training | healthy |
| GSM1146429 | training | healthy |
| GSM1146353 | training | healthy |
| GSM1146354 | training | healthy |
| GSM1146355 | training | healthy |
| GSM2641325 | training | healthy |
| GSM2641326 | training | healthy |
| GSM2641327 | training | healthy |
| GSM2641153 | training | healthy |
| GSM2641154 | training | healthy |
| GSM2641155 | training | healthy |
| GSM2641156 | training | healthy |
| GSM2811043 | training | healthy |
| GSM2811044 | training | healthy |
| GSM2811045 | training | healthy |
| GSM2811046 | training | healthy |
| GSM2811047 | training | healthy |
| GSM2811048 | training | healthy |
| GSM2811049 | training | healthy |
| GSM2811050 | training | healthy |
| GSM2811051 | training | healthy |
| GSM2811052 | training | healthy |
| GSM2811053 | training | healthy |
| GSM2811054 | training | healthy |
| GSM2811055 | training | healthy |
| GSM2811056 | training | healthy |
| GSM2811057 | training | healthy |
| GSM2811058 | training | healthy |
| GSM2811059 | training | healthy |
| GSM2811060 | training | healthy |
| GSM757014 | training | diseased |
| GSM757015 | training | diseased |
| GSM757016 | training | diseased |
| GSM757017 | training | diseased |
| GSM757018 | training | diseased |
| GSM757019 | training | diseased |
| GSM757020 | training | diseased |
| GSM757021 | training | diseased |
| GSM757022 | training | diseased |
| GSM757023 | training | diseased |
| GSM1146387 | training | diseased |
| GSM1146388 | training | diseased |
| GSM1146389 | training | diseased |
| GSM1146390 | training | diseased |
| GSM1146391 | training | diseased |
| GSM1146392 | training | diseased |
| GSM1146393 | training | diseased |
| GSM1146327 | training | diseased |
| GSM1146328 | training | diseased |
| GSM1146329 | training | diseased |
| GSM1146330 | training | diseased |
| GSM1146331 | training | diseased |
| GSM1146332 | training | diseased |
| GSM1146333 | training | diseased |
| GSM1146334 | training | diseased |
| GSM1146335 | training | diseased |
| GSM1146336 | training | diseased |
| GSM1146337 | training | diseased |
| GSM2641157 | training | diseased |
| GSM2641158 | training | diseased |
| GSM2641159 | training | diseased |
| GSM2641160 | training | diseased |
| GSM2641161 | training | diseased |
| GSM2641162 | training | diseased |
| GSM2641163 | training | diseased |
| GSM2641164 | training | diseased |
| GSM2641165 | training | diseased |
| GSM2641166 | training | diseased |
| GSM2641167 | training | diseased |
| GSM2641285 | training | diseased |
| GSM2641286 | training | diseased |
| GSM2641287 | training | diseased |
| GSM2641288 | training | diseased |
| GSM2641289 | training | diseased |
| GSM2641290 | training | diseased |
| GSM2641291 | training | diseased |
| GSM2811029 | training | diseased |
| GSM2811030 | training | diseased |
| GSM2811031 | training | diseased |
| GSM2811032 | training | diseased |
| GSM2811033 | training | diseased |
| GSM2811034 | training | diseased |
| GSM2811035 | training | diseased |
| GSM2810894 | training | diseased |
| GSM2810895 | training | diseased |
| GSM2810896 | training | diseased |
| GSM2810897 | training | diseased |
| GSM2810898 | training | diseased |
| GSM2810899 | training | diseased |
| GSM2810900 | training | diseased |
| GSM2810901 | training | diseased |
| GSM2810902 | training | diseased |
| GSM2810903 | training | diseased |
| GSM2544316 | validation | healthy |
| GSM2544317 | validation | healthy |
| GSM2544318 | validation | healthy |
| GSM2544319 | validation | healthy |
| GSM2544320 | validation | healthy |
| GSM2544321 | validation | healthy |
| GSM2544322 | validation | healthy |
| GSM2544323 | validation | healthy |
| GSM2544324 | validation | healthy |
| GSM2544325 | validation | healthy |
| GSM2544326 | validation | healthy |
| GSM2544327 | validation | healthy |
| GSM2544328 | validation | healthy |
| GSM2544329 | validation | healthy |
| GSM2544330 | validation | healthy |
| GSM2544331 | validation | healthy |
| GSM2544332 | validation | healthy |
| GSM2544333 | validation | healthy |
| GSM2544334 | validation | healthy |
| GSM2544335 | validation | healthy |
| GSM2544275 | validation | diseased |
| GSM2544276 | validation | diseased |
| GSM2544277 | validation | diseased |
| GSM2544278 | validation | diseased |
| GSM2544279 | validation | diseased |
| GSM2544280 | validation | diseased |
| GSM2544281 | validation | diseased |
| GSM2544282 | validation | diseased |
| GSM2544283 | validation | diseased |
| GSM2544284 | validation | diseased |
| GSM2544285 | validation | diseased |
| GSM2544286 | validation | diseased |
| GSM2544287 | validation | diseased |
| GSM2544288 | validation | diseased |
| GSM2544289 | validation | diseased |
| GSM2544290 | validation | diseased |
| GSM2544291 | validation | diseased |
| GSM2544292 | validation | diseased |
| GSM2544293 | validation | diseased |
| GSM2544294 | validation | diseased |
| GSM2544295 | validation | diseased |
| GSM2544296 | validation | diseased |
| GSM2544297 | validation | diseased |
| GSM2544298 | validation | diseased |
| GSM2544299 | validation | diseased |
| GSM2544300 | validation | diseased |
| GSM2544301 | validation | diseased |
| GSM2544302 | validation | diseased |
| GSM2544303 | validation | diseased |
| GSM2544304 | validation | diseased |
| GSM2544305 | validation | diseased |
| GSM2544306 | validation | diseased |
| GSM2544307 | validation | diseased |
| GSM2544308 | validation | diseased |
| GSM2544309 | validation | diseased |
| GSM2544310 | validation | diseased |
| GSM2544311 | validation | diseased |
| GSM2544312 | validation | diseased |
| GSM2544313 | validation | diseased |
| GSM2544314 | validation | diseased |
| GSM2544315 | validation | diseased |
