# Supplementary material for: Identification of immune-associated biomarkers of diabetes nephropathy tubulointerstitial injury based on machine learning: a bioinformatics multi-chip integrated analysis
Source: BioData Min. 2024 Jul 1;17:20. doi: 10.1186/s13040-024-00369-x (PMC11218417; doi:10.1186/s13040-024-00369-x)
Supplement: Supplementary file 4 — Supplementary Material 4 [file 13040_2024_369_MOESM4_ESM.docx]

**Supplementary TABLE 5:** LASSO algorithm screening characteristic genes for DN.

|  | **Gene** | **Coef** |
| --- | --- | --- |
| 1 | FSTL1 | 1.312775296 |
| 2 | MMP7 | 0.479199578 |
| 3 | CD53 | 1.008654192 |
| 4 | NR4A1 | -0.673483217 |
| 5 | ARG2 | -1.016921213 |
| 6 | CX3CR1 | 0.081280668 |
| 7 | AGR2 | 1.975297529 |
| 8 | VIM | 0.274484081 |
| 9 | S100A8 | -0.754640001 |
| 10 | ITGAM | 0.260083904 |
| 11 | JUN | -0.507146718 |
| 12 | CEBPD | -3.000273692 |
| 13 | ZFP36 | -0.321178316 |
| 14 | NXN | 0.093376698 |
| 15 | PLSCR1 | 1.291289989 |
| 16 | REG1A | -0.895368859 |
| 17 | NR0B2 | -1.217552295 |
| 18 | CLEC10A | -2.115578415 |
| 19 | DEFB1 | -1.914778239 |
| 20 | CCR2 | 1.422480263 |
| 21 | DDX60 | 0.866963795 |
| 22 | CISH | -2.784888576 |
