# Supplementary material for: Identification of immune-associated biomarkers of diabetes nephropathy tubulointerstitial injury based on machine learning: a bioinformatics multi-chip integrated analysis
Source: BioData Min. 2024 Jul 1;17:20. doi: 10.1186/s13040-024-00369-x (PMC11218417; doi:10.1186/s13040-024-00369-x)
Supplement: Supplementary file 5 — Supplementary Material 5 [file 13040_2024_369_MOESM5_ESM.docx]

**Supplementary TABLE 4:** The 119 Immune-Associated Biomarkers and their differential expression characteristics.

| id | logFC | AveExpr | P.Value | adj.P.Val |
| --- | --- | --- | --- | --- |
| PTGER3 | -0.578527067 | 4.883928035 | 2.14E-16 | 6.72E-13 |
| CISH | -0.661281333 | 5.682121659 | 6.90E-16 | 1.62E-12 |
| NR4A1 | -0.673397597 | 4.182930836 | 4.06E-15 | 5.62E-12 |
| ANXA1 | 1.582207375 | 5.20590551 | 1.67E-14 | 1.22E-11 |
| CX3CR1 | 1.268256561 | 4.555913632 | 2.74E-14 | 1.72E-11 |
| DEFB1 | -0.901621136 | 8.387351447 | 3.28E-14 | 1.93E-11 |
| VIM | 1.416799324 | 7.492814198 | 5.60E-14 | 3.10E-11 |
| FSTL1 | 1.080337777 | 6.347789639 | 1.34E-13 | 6.64E-11 |
| AGR2 | 1.040276112 | 4.18169387 | 1.74E-13 | 8.14E-11 |
| ZFP36 | -0.84984309 | 5.811173505 | 3.92E-13 | 1.53E-10 |
| KLK1 | -1.281548022 | 5.506950587 | 4.06E-13 | 1.53E-10 |
| NR0B2 | -0.608025429 | 4.524163942 | 5.90E-13 | 2.06E-10 |
| ANXA2 | 1.222473033 | 7.485853141 | 1.05E-12 | 3.08E-10 |
| DUSP1 | -0.821791133 | 5.183721786 | 3.43E-12 | 7.37E-10 |
| MMP7 | 1.749587486 | 7.219491321 | 3.45E-12 | 7.37E-10 |
| HRG | -0.988444864 | 5.705708703 | 5.13E-12 | 1.05E-09 |
| ARG2 | -0.611744447 | 5.882273674 | 5.91E-12 | 1.11E-09 |
| IFI16 | 1.096828986 | 4.428189014 | 6.40E-12 | 1.16E-09 |
| ADM | -1.01682199 | 6.204279141 | 1.29E-11 | 1.93E-09 |
| LYZ | 2.430652675 | 4.546459566 | 1.64E-11 | 2.24E-09 |
| NXN | 0.796276175 | 4.389914828 | 2.51E-11 | 3.22E-09 |
| TRIM22 | 1.14169307 | 5.508579432 | 2.66E-11 | 3.22E-09 |
| DCK | 0.810624478 | 3.703661871 | 8.06E-11 | 7.50E-09 |
| EGF | -1.378449249 | 5.569855406 | 8.06E-11 | 7.50E-09 |
| TNC | 1.044136746 | 4.500546341 | 8.12E-11 | 7.50E-09 |
| LUM | 1.341501549 | 6.24855426 | 9.67E-11 | 8.35E-09 |
| USP2 | -0.855648916 | 4.68692162 | 1.04E-10 | 8.88E-09 |
| CD53 | 1.002938623 | 5.716573156 | 1.55E-10 | 1.25E-08 |
| TMSB10 | 1.000790722 | 8.212809172 | 1.73E-10 | 1.36E-08 |
| LY96 | 1.549540855 | 3.58428119 | 1.92E-10 | 1.48E-08 |
| LYN | 0.573675722 | 4.159854501 | 1.98E-10 | 1.48E-08 |
| SLIT2 | -0.650443539 | 5.707961422 | 5.26E-10 | 3.42E-08 |
| LTF | 2.200603564 | 6.225108368 | 8.66E-10 | 5.06E-08 |
| CD48 | 1.134405793 | 4.862171179 | 1.15E-09 | 6.16E-08 |
| ARPC5 | 0.567667333 | 6.541032333 | 1.26E-09 | 6.53E-08 |
| CASP1 | 0.909290236 | 3.749386871 | 2.01E-09 | 9.46E-08 |
| CCR2 | 0.967900146 | 3.068770183 | 2.72E-09 | 1.17E-07 |
| PYCARD | 1.136914986 | 3.775120422 | 2.88E-09 | 1.22E-07 |
| HLA-DPA1 | 1.066925343 | 7.05682714 | 3.24E-09 | 1.34E-07 |
| RORA | -0.604654881 | 4.482078519 | 3.59E-09 | 1.47E-07 |
| IFITM2 | 0.880298985 | 6.59127592 | 4.55E-09 | 1.74E-07 |
| CRLF3 | 0.539276374 | 4.860576662 | 5.98E-09 | 2.14E-07 |
| TNFRSF11B | 0.606818821 | 5.533524962 | 8.12E-09 | 2.68E-07 |
| C1S | 1.139579692 | 5.507176097 | 1.20E-08 | 3.64E-07 |
| DDX60 | 0.641359305 | 4.557682585 | 1.43E-08 | 4.21E-07 |
| LY86 | 0.630168109 | 4.814838805 | 1.50E-08 | 4.36E-07 |
| ITGAM | 0.660092627 | 3.562850155 | 1.54E-08 | 4.45E-07 |
| CTSS | 0.879485098 | 3.76423314 | 1.56E-08 | 4.51E-07 |
| CFD | 0.704169868 | 4.130736304 | 1.94E-08 | 5.37E-07 |
| PLSCR1 | 0.709390431 | 6.384443054 | 2.15E-08 | 5.79E-07 |
| IGKC | 1.444204485 | 7.841902796 | 2.30E-08 | 6.11E-07 |
| CEBPD | -0.523445763 | 7.129169684 | 2.38E-08 | 6.26E-07 |
| ITGB2 | 0.787905979 | 4.356481192 | 2.61E-08 | 6.72E-07 |
| LGALS1 | 0.942298893 | 6.010177052 | 3.36E-08 | 8.26E-07 |
| A2M | 0.777812615 | 7.052851098 | 3.70E-08 | 8.94E-07 |
| CYFIP2 | -0.530542374 | 6.441012776 | 4.07E-08 | 9.69E-07 |
| HLA-DRA | 0.867066545 | 7.203461965 | 5.05E-08 | 1.14E-06 |
| PPARGC1A | -0.516841684 | 5.433123284 | 6.13E-08 | 1.32E-06 |
| GMFG | 0.766728215 | 4.723357333 | 6.29E-08 | 1.35E-06 |
| CAV1 | 0.651216962 | 4.461979204 | 8.26E-08 | 1.69E-06 |
| TYROBP | 1.023168638 | 5.102645633 | 8.29E-08 | 1.69E-06 |
| RAC2 | 0.626420549 | 4.862934489 | 1.00E-07 | 1.96E-06 |
| HLA-DPB1 | 0.90873956 | 6.232472745 | 1.09E-07 | 2.11E-06 |
| WFDC2 | 0.987973351 | 6.253792956 | 1.29E-07 | 2.42E-06 |
| C3 | 1.296517534 | 6.38595391 | 1.48E-07 | 2.73E-06 |
| POSTN | 1.207988673 | 4.355998715 | 1.51E-07 | 2.76E-06 |
| NFIL3 | -0.614742121 | 5.603308066 | 1.53E-07 | 2.79E-06 |
| KNG1 | -0.799627061 | 6.078639133 | 1.55E-07 | 2.81E-06 |
| CD1C | 0.507726905 | 4.755039515 | 1.79E-07 | 3.16E-06 |
| CKLF | 0.612965857 | 4.459823074 | 1.86E-07 | 3.24E-06 |
| ATF3 | -1.039546249 | 4.401132571 | 3.82E-07 | 5.80E-06 |
| PTPRC | 0.869702636 | 3.454439856 | 4.67E-07 | 6.80E-06 |
| CFH | 0.864804196 | 4.31953875 | 4.85E-07 | 6.98E-06 |
| CLU | 0.854104533 | 6.538419236 | 4.90E-07 | 7.03E-06 |
| LTB | 0.688566469 | 4.625589735 | 5.30E-07 | 7.50E-06 |
| HLA-B | 0.633103015 | 8.137437621 | 7.59E-07 | 9.97E-06 |
| IRF8 | 0.726814428 | 5.089280766 | 7.86E-07 | 1.02E-05 |
| EGR1 | -0.838550071 | 5.046395141 | 8.71E-07 | 1.11E-05 |
| NCF2 | 0.758482782 | 3.456560507 | 1.01E-06 | 1.25E-05 |
| IL10RA | 0.691637385 | 4.017488047 | 1.35E-06 | 1.56E-05 |
| PSMB8 | 0.587832035 | 5.270174357 | 1.49E-06 | 1.68E-05 |
| CD3D | 0.695421986 | 4.065629785 | 1.55E-06 | 1.73E-05 |
| FCER1G | 0.628241919 | 5.398776919 | 1.85E-06 | 2.00E-05 |
| CCL19 | 1.211078626 | 4.117239229 | 2.17E-06 | 2.27E-05 |
| RFTN1 | 0.591282433 | 5.307701375 | 2.19E-06 | 2.28E-05 |
| FOS | -1.339577664 | 4.605837224 | 3.01E-06 | 2.97E-05 |
| IFI27 | 0.762343776 | 5.716463485 | 4.17E-06 | 3.89E-05 |
| S100A11 | 0.579666552 | 6.574530218 | 6.10E-06 | 5.35E-05 |
| C1QB | 0.887221789 | 4.606519024 | 6.12E-06 | 5.35E-05 |
| HLA-F | 0.492994284 | 6.960192814 | 7.40E-06 | 6.18E-05 |
| C1QA | 0.772378465 | 4.771777266 | 8.98E-06 | 7.23E-05 |
| VCAM1 | 0.6055724 | 7.634592374 | 1.03E-05 | 8.12E-05 |
| C1R | 0.759300677 | 5.453050642 | 1.23E-05 | 9.34E-05 |
| FGF9 | -0.513350132 | 4.966697684 | 2.07E-05 | 0.000144411 |
| HLA-DMA | 0.599697685 | 5.975146808 | 2.58E-05 | 0.000171958 |
| CXCL12 | 0.499269604 | 6.301602557 | 2.77E-05 | 0.000182516 |
| IGHM | 0.807114299 | 4.73673712 | 3.86E-05 | 0.00024135 |
| ISG20 | 0.579566617 | 3.739834631 | 3.87E-05 | 0.000242028 |
| FCN1 | 0.624383193 | 3.353257617 | 5.24E-05 | 0.000307603 |
| FCGR2B | 0.604839522 | 3.565281772 | 5.31E-05 | 0.000310455 |
| TRAF5 | 0.507722147 | 3.6645078 | 5.50E-05 | 0.000319215 |
| JUN | -0.527369287 | 4.757646748 | 0.000128934 | 0.000643988 |
| CCL5 | 0.691845163 | 3.576366905 | 0.000135328 | 0.000668827 |
| C7 | 0.621253133 | 6.916980814 | 0.000189703 | 0.000883746 |
| SERPINA3 | 0.959499647 | 5.981556791 | 0.000190402 | 0.000886565 |
| PLA2G4A | 0.679372212 | 2.987037613 | 0.000194553 | 0.000903214 |
| CXCL1 | 0.685754355 | 3.348529075 | 0.000199546 | 0.000922752 |
| GDF15 | -0.67007935 | 5.697248121 | 0.000236443 | 0.001060051 |
| PLTP | 0.567633788 | 5.171874215 | 0.000329312 | 0.001399602 |
| BST2 | 0.539276805 | 5.095759961 | 0.000519521 | 0.002026218 |
| HLA-DQA1 | 0.909539328 | 4.629481311 | 0.001295731 | 0.004332141 |
| CLEC10A | 0.501716336 | 3.597753734 | 0.00129648 | 0.004333104 |
| DCN | 0.546819077 | 6.500349723 | 0.001814072 | 0.005713368 |
| CXCL9 | 0.508202763 | 4.12671878 | 0.001858228 | 0.005835631 |
| MAFB | 0.520171803 | 4.953073171 | 0.003807278 | 0.010558327 |
| HLA-DQB1 | 0.593383503 | 5.043786716 | 0.004944137 | 0.013068235 |
| OLFM4 | 0.59973811 | 3.618217803 | 0.028155938 | 0.056509946 |
| S100A8 | 0.596523366 | 4.629451902 | 0.043742703 | 0.081358663 |
| REG1A | 0.462951269 | 4.282882383 | 0.079343055 | 0.133110275 |
