# Supplementary material for: Identification of immune-associated biomarkers of diabetes nephropathy tubulointerstitial injury based on machine learning: a bioinformatics multi-chip integrated analysis
Source: BioData Min. 2024 Jul 1;17:20. doi: 10.1186/s13040-024-00369-x (PMC11218417; doi:10.1186/s13040-024-00369-x)
Supplement: Supplementary file 6 — Supplementary Material 6 [file 13040_2024_369_MOESM6_ESM.docx]

**Supplementary TABLE 3:** The 454 DEGs and their differential expression characteristics.

| id | logFC | AveExpr | P.Value | adj.P.Val |
| --- | --- | --- | --- | --- |
| PTGER3 | -0.575222182 | 4.886440714 | 2.00E-16 | 6.80E-13 |
| GADD45B | -1.125376905 | 5.549114157 | 2.17E-16 | 6.80E-13 |
| CX3CR1 | 1.279747919 | 4.572537255 | 1.33E-15 | 3.14E-12 |
| MARCKSL1 | 0.897345657 | 5.471984913 | 3.80E-15 | 4.48E-12 |
| PROM1 | 1.759932154 | 6.058733049 | 7.13E-15 | 6.74E-12 |
| CISH | -0.651479228 | 5.682915856 | 8.00E-15 | 6.74E-12 |
| MYOF | 0.797555438 | 5.309599016 | 8.39E-15 | 6.74E-12 |
| ANXA1 | 1.568531299 | 5.219738885 | 8.59E-15 | 6.74E-12 |
| NR4A1 | -0.666276757 | 4.182621572 | 1.12E-14 | 8.08E-12 |
| FSTL1 | 1.105258649 | 6.342752701 | 2.57E-14 | 1.73E-11 |
| DLST | -0.5759595 | 6.104633072 | 3.32E-14 | 2.09E-11 |
| ALOX5 | 0.599257195 | 3.390517468 | 7.93E-14 | 4.18E-11 |
| VIM | 1.437061246 | 7.497811238 | 7.99E-14 | 4.18E-11 |
| AGR2 | 1.004789686 | 4.199029161 | 9.94E-14 | 4.93E-11 |
| DEFB1 | -0.870179766 | 8.386106611 | 1.33E-13 | 6.28E-11 |
| GADD45A | -0.851246461 | 7.154181642 | 1.60E-13 | 7.17E-11 |
| VCAN | 1.399098444 | 3.86921879 | 2.23E-13 | 9.13E-11 |
| CEL | -0.808614108 | 3.599526701 | 3.49E-13 | 1.37E-10 |
| ANXA2 | 1.26647413 | 7.4936408 | 4.15E-13 | 1.56E-10 |
| TIMP1 | 1.519434927 | 6.160374432 | 6.42E-13 | 2.12E-10 |
| TPM1 | 0.751440417 | 6.446606045 | 7.62E-13 | 2.33E-10 |
| COL4A1 | 0.877609439 | 6.095541969 | 7.87E-13 | 2.33E-10 |
| KLK1 | -1.265844837 | 5.508283178 | 8.63E-13 | 2.46E-10 |
| KLF9 | -0.603464153 | 4.754961332 | 1.36E-12 | 3.76E-10 |
| ZFP36 | -0.836924345 | 5.806897802 | 1.45E-12 | 3.81E-10 |
| MMP7 | 1.795722586 | 7.22409394 | 1.46E-12 | 3.81E-10 |
| NR0B2 | -0.594713582 | 4.51679058 | 1.63E-12 | 4.15E-10 |
| IFI16 | 1.023738366 | 4.462390486 | 1.80E-12 | 4.27E-10 |
| LYZ | 2.229794009 | 4.721073646 | 1.81E-12 | 4.27E-10 |
| KDELR3 | 0.537945756 | 4.093105637 | 2.01E-12 | 4.50E-10 |
| TSPAN13 | 0.87925755 | 5.604091782 | 2.57E-12 | 5.64E-10 |
| TNFAIP8 | 1.041607045 | 5.02577347 | 2.66E-12 | 5.70E-10 |
| DCK | 0.693514548 | 3.764753296 | 2.76E-12 | 5.77E-10 |
| G0S2 | -0.948376778 | 6.014967645 | 2.92E-12 | 5.98E-10 |
| CASP1 | 0.826929113 | 3.839053281 | 4.00E-12 | 7.84E-10 |
| PDLIM1 | 0.796279254 | 6.74734038 | 4.15E-12 | 7.97E-10 |
| SPRY1 | 0.568086088 | 5.058844758 | 4.39E-12 | 8.11E-10 |
| PLK2 | 1.048651148 | 3.69319112 | 4.39E-12 | 8.11E-10 |
| CLEC2B | 0.833569027 | 4.666741994 | 6.89E-12 | 1.10E-09 |
| TUSC3 | 0.742308529 | 4.948655793 | 7.26E-12 | 1.14E-09 |
| DUSP1 | -0.810570994 | 5.185073304 | 9.41E-12 | 1.36E-09 |
| CD44 | 0.572983414 | 4.224693549 | 1.26E-11 | 1.70E-09 |
| SLC2A10 | 0.721590187 | 3.333784597 | 1.39E-11 | 1.82E-09 |
| PVALB | -1.168925223 | 5.148698343 | 1.39E-11 | 1.82E-09 |
| HRG | -0.982372122 | 5.705546508 | 1.49E-11 | 1.92E-09 |
| TRIM22 | 1.160384284 | 5.508680282 | 1.71E-11 | 2.13E-09 |
| COL3A1 | 1.486945824 | 5.272494142 | 1.79E-11 | 2.19E-09 |
| ARL6IP5 | 0.532893329 | 6.888818753 | 2.08E-11 | 2.51E-09 |
| TSKU | -0.691460805 | 5.674322399 | 2.14E-11 | 2.56E-09 |
| NXN | 0.800231501 | 4.389303818 | 2.18E-11 | 2.57E-09 |
| ARG2 | -0.597017657 | 5.882582191 | 3.22E-11 | 3.52E-09 |
| PDK4 | -0.934171975 | 5.065577785 | 3.40E-11 | 3.64E-09 |
| VWF | 0.559695463 | 4.278557245 | 3.49E-11 | 3.69E-09 |
| SH2B3 | 0.634533865 | 5.188777886 | 3.65E-11 | 3.80E-09 |
| ADM | -0.998255267 | 6.199778374 | 3.72E-11 | 3.80E-09 |
| TGFBI | 1.041158712 | 5.588798914 | 3.76E-11 | 3.80E-09 |
| USP2 | -0.838956873 | 4.698001102 | 4.67E-11 | 4.58E-09 |
| TMSB10 | 1.011761002 | 8.213718881 | 5.08E-11 | 4.93E-09 |
| LY96 | 1.267409622 | 3.77478907 | 5.56E-11 | 5.33E-09 |
| CD53 | 1.023117536 | 5.712278675 | 5.86E-11 | 5.41E-09 |
| CPA3 | 1.327034463 | 4.559706738 | 5.95E-11 | 5.43E-09 |
| CCR2 | 0.565864394 | 3.370111969 | 6.26E-11 | 5.63E-09 |
| LUM | 1.35391217 | 6.245401742 | 6.94E-11 | 6.05E-09 |
| RRM2 | 0.539013792 | 3.404878425 | 7.04E-11 | 6.05E-09 |
| ANXA3 | 0.959964636 | 4.742543613 | 7.19E-11 | 6.05E-09 |
| CTSS | 0.783271828 | 3.863258759 | 7.21E-11 | 6.05E-09 |
| TNC | 1.049188027 | 4.50384625 | 7.26E-11 | 6.05E-09 |
| AP1S2 | 0.576808629 | 4.709414695 | 7.80E-11 | 6.44E-09 |
| EGF | -1.345697941 | 5.584904502 | 9.09E-11 | 7.31E-09 |
| MYLIP | 0.523178632 | 5.108327015 | 9.52E-11 | 7.47E-09 |
| PYCARD | 0.969136363 | 3.895754219 | 1.28E-10 | 9.56E-09 |
| QPCT | 0.971695919 | 3.910615327 | 1.39E-10 | 1.01E-08 |
| PLAC8 | 1.03838905 | 3.639338254 | 1.39E-10 | 1.01E-08 |
| DEK | 0.51049087 | 6.644301752 | 1.48E-10 | 1.07E-08 |
| LYN | 0.574650016 | 4.164260129 | 1.54E-10 | 1.10E-08 |
| MACROD1 | -0.782047278 | 5.206125034 | 1.59E-10 | 1.12E-08 |
| ACTN1 | 0.785095653 | 5.137514619 | 1.74E-10 | 1.23E-08 |
| C11orf71 | -0.609016361 | 5.113447591 | 1.80E-10 | 1.26E-08 |
| MAPT | -0.553976954 | 5.190477044 | 1.88E-10 | 1.30E-08 |
| RHCG | -0.947085731 | 5.553094466 | 2.23E-10 | 1.51E-08 |
| ITGAM | 0.60653007 | 3.619274587 | 2.24E-10 | 1.51E-08 |
| PLP2 | 0.800040887 | 5.268838421 | 2.52E-10 | 1.69E-08 |
| ISOC2 | -0.6281014 | 6.237147028 | 2.98E-10 | 1.96E-08 |
| EVI2B | 0.93706059 | 3.475901981 | 3.38E-10 | 2.21E-08 |
| TUBA1A | 0.923804847 | 7.036444506 | 3.45E-10 | 2.23E-08 |
| FGL2 | 0.87136524 | 6.051113685 | 3.64E-10 | 2.32E-08 |
| THBS2 | 1.264262777 | 5.232092816 | 3.71E-10 | 2.34E-08 |
| SGK2 | -0.555535618 | 5.321455971 | 3.80E-10 | 2.38E-08 |
| LTF | 2.259934694 | 6.252928989 | 3.94E-10 | 2.43E-08 |
| CRIP1 | 0.808029377 | 4.737247718 | 4.21E-10 | 2.54E-08 |
| FHL1 | 0.72164085 | 4.681287233 | 4.44E-10 | 2.66E-08 |
| SCRN1 | 0.631558065 | 5.266375736 | 4.92E-10 | 2.89E-08 |
| CSTA | 0.956257586 | 3.467872744 | 5.03E-10 | 2.94E-08 |
| ASB9 | -0.711344247 | 4.875162997 | 5.07E-10 | 2.95E-08 |
| FN1 | 0.550940783 | 4.399364421 | 5.50E-10 | 3.16E-08 |
| MANSC1 | 0.640100324 | 4.017848306 | 6.58E-10 | 3.73E-08 |
| MS4A4A | 0.890423552 | 3.815544977 | 6.64E-10 | 3.74E-08 |
| EYA2 | -0.538949491 | 4.063036501 | 6.84E-10 | 3.81E-08 |
| PECAM1 | 0.62126966 | 5.033410948 | 6.96E-10 | 3.85E-08 |
| EVI2A | 1.190862046 | 3.907871998 | 7.39E-10 | 4.02E-08 |
| TMPRSS4 | 0.577568163 | 4.893686266 | 7.49E-10 | 4.03E-08 |
| PON2 | 0.741111896 | 5.987476156 | 7.98E-10 | 4.22E-08 |
| FOSB | -1.443012803 | 4.019085204 | 8.47E-10 | 4.45E-08 |
| CD48 | 1.142498752 | 4.861514884 | 8.56E-10 | 4.45E-08 |
| HLA-DPA1 | 1.103304662 | 7.05729883 | 8.56E-10 | 4.45E-08 |
| ARPC5 | 0.58142233 | 6.542246999 | 8.60E-10 | 4.45E-08 |
| TMEM45A | 0.733405437 | 2.989387851 | 9.00E-10 | 4.58E-08 |
| COL1A2 | 1.176790637 | 4.880387003 | 9.28E-10 | 4.67E-08 |
| APOLD1 | -0.791613227 | 3.817304269 | 1.02E-09 | 5.05E-08 |
| RALYL | -0.902244643 | 5.252783864 | 1.04E-09 | 5.08E-08 |
| RNASET2 | 0.55304016 | 5.86234824 | 1.13E-09 | 5.43E-08 |
| ITM2C | 0.710803066 | 6.427024216 | 1.14E-09 | 5.50E-08 |
| LAPTM5 | 1.072546775 | 5.36261936 | 1.21E-09 | 5.77E-08 |
| PTPRC | 0.750092338 | 3.629091684 | 1.37E-09 | 6.37E-08 |
| FXYD5 | 0.649820208 | 4.36646786 | 1.42E-09 | 6.49E-08 |
| COL6A3 | 1.251041448 | 4.597215561 | 1.43E-09 | 6.51E-08 |
| ESYT1 | 0.586137791 | 5.561318871 | 1.47E-09 | 6.61E-08 |
| AVPI1 | -0.520722852 | 4.819597692 | 1.73E-09 | 7.65E-08 |
| SLIT2 | -0.640719805 | 5.71284329 | 1.75E-09 | 7.68E-08 |
| TPBG | 0.844753649 | 4.927313013 | 1.80E-09 | 7.90E-08 |
| CHST15 | 0.649267791 | 4.164894534 | 1.84E-09 | 7.99E-08 |
| NELL1 | -0.622868032 | 4.316960195 | 1.94E-09 | 8.27E-08 |
| COL4A2 | 0.625525779 | 5.632900481 | 1.96E-09 | 8.29E-08 |
| RTP4 | 0.608641767 | 3.927665117 | 2.01E-09 | 8.37E-08 |
| EFNB2 | 0.530545973 | 4.313835436 | 2.06E-09 | 8.55E-08 |
| CRIP2 | 0.530197947 | 5.035061493 | 2.23E-09 | 9.10E-08 |
| METTL1 | -0.586928106 | 5.530035214 | 2.57E-09 | 1.04E-07 |
| IFITM2 | 0.901634686 | 6.590285564 | 2.62E-09 | 1.05E-07 |
| HYAL1 | -0.618107791 | 6.642303608 | 2.62E-09 | 1.05E-07 |
| DDX60 | 0.622052341 | 4.571389958 | 2.91E-09 | 1.14E-07 |
| CSDC2 | -0.558512469 | 4.94771957 | 3.00E-09 | 1.17E-07 |
| PDK2 | -0.532316502 | 5.455471478 | 3.06E-09 | 1.19E-07 |
| LIPA | 0.572860068 | 6.618182722 | 3.34E-09 | 1.26E-07 |
| YWHAH | 0.592593129 | 5.438939177 | 3.47E-09 | 1.30E-07 |
| RORA | -0.605412979 | 4.487949536 | 3.84E-09 | 1.40E-07 |
| LPCAT1 | 0.665295228 | 4.709801267 | 3.86E-09 | 1.40E-07 |
| SH3GL2 | -0.823876473 | 5.02522672 | 3.89E-09 | 1.41E-07 |
| GIMAP4 | 0.602940878 | 5.048061396 | 4.18E-09 | 1.49E-07 |
| BHLHE40 | -0.500837119 | 5.421476212 | 4.27E-09 | 1.51E-07 |
| SLC19A2 | -0.669710283 | 4.697012806 | 4.44E-09 | 1.56E-07 |
| RGS10 | 0.760252825 | 4.428943217 | 4.92E-09 | 1.71E-07 |
| CHODL | 0.670905562 | 4.329029194 | 4.97E-09 | 1.71E-07 |
| RNASE1 | 0.659825751 | 6.815613326 | 5.26E-09 | 1.80E-07 |
| GNG11 | 0.67369744 | 5.673713295 | 5.77E-09 | 1.93E-07 |
| GADD45G | -0.689872963 | 3.789466911 | 6.03E-09 | 2.00E-07 |
| IFITM1 | 1.03550089 | 6.455970104 | 6.39E-09 | 2.08E-07 |
| KRT19 | 1.021145557 | 5.740587303 | 6.42E-09 | 2.08E-07 |
| FLRT3 | 0.765596504 | 6.083706436 | 6.45E-09 | 2.08E-07 |
| HOPX | 1.143931182 | 4.495509695 | 6.46E-09 | 2.08E-07 |
| C1S | 1.161547497 | 5.5003968 | 6.58E-09 | 2.09E-07 |
| TES | 0.608915256 | 4.850079851 | 6.79E-09 | 2.14E-07 |
| MEST | 0.724043821 | 6.106425697 | 7.10E-09 | 2.22E-07 |
| CRLF3 | 0.538402303 | 4.858779521 | 7.29E-09 | 2.26E-07 |
| TFPI2 | 1.19531775 | 4.200607136 | 7.78E-09 | 2.40E-07 |
| CXADR | 0.786038453 | 5.089792594 | 8.67E-09 | 2.61E-07 |
| GZMA | 0.89867339 | 3.792666158 | 8.81E-09 | 2.63E-07 |
| LY86 | 0.639375433 | 4.808954233 | 8.86E-09 | 2.63E-07 |
| GLB1L2 | -0.622499065 | 6.138806431 | 9.68E-09 | 2.86E-07 |
| DOCK2 | 0.533648886 | 3.313813303 | 1.16E-08 | 3.32E-07 |
| MT1X | -0.861201482 | 8.132040907 | 1.17E-08 | 3.36E-07 |
| FZD6 | 0.546276405 | 5.085319765 | 1.19E-08 | 3.36E-07 |
| A2M | 0.794687449 | 7.048733307 | 1.19E-08 | 3.36E-07 |
| IFI44 | 0.639609297 | 3.843428502 | 1.23E-08 | 3.45E-07 |
| IGKC | 1.491067709 | 7.860920752 | 1.29E-08 | 3.58E-07 |
| CALML3 | -0.762267985 | 4.243821376 | 1.31E-08 | 3.64E-07 |
| RAB31 | 0.680617139 | 4.628326953 | 1.32E-08 | 3.65E-07 |
| SPATA2L | -0.542824052 | 5.107626378 | 1.34E-08 | 3.68E-07 |
| LOXL1 | 0.809665517 | 4.159837693 | 1.34E-08 | 3.68E-07 |
| LGALS1 | 0.966228254 | 6.007334513 | 1.44E-08 | 3.85E-07 |
| TNFRSF11B | 0.606101219 | 5.542067249 | 1.63E-08 | 4.29E-07 |
| PLSCR1 | 0.728667865 | 6.385878531 | 1.65E-08 | 4.34E-07 |
| COL15A1 | 0.741248635 | 4.066690695 | 1.71E-08 | 4.45E-07 |
| FHL2 | 0.877086393 | 5.342417832 | 1.71E-08 | 4.45E-07 |
| NMI | 0.595347819 | 5.092040598 | 1.80E-08 | 4.62E-07 |
| ARHGDIB | 0.928038665 | 5.454027912 | 1.81E-08 | 4.64E-07 |
| NCF2 | 0.617469426 | 3.595690722 | 2.05E-08 | 5.18E-07 |
| CFD | 0.671796477 | 4.142836739 | 2.11E-08 | 5.27E-07 |
| ITGB2 | 0.791373919 | 4.357928143 | 2.12E-08 | 5.28E-07 |
| MARCKS | 0.592316539 | 4.064448781 | 2.19E-08 | 5.35E-07 |
| ACOT7 | -0.640684897 | 6.28724424 | 2.29E-08 | 5.59E-07 |
| NPY1R | 0.855616748 | 6.002991351 | 2.84E-08 | 6.71E-07 |
| HTR2B | 0.770721627 | 3.360925176 | 2.84E-08 | 6.71E-07 |
| EAF2 | -0.71390894 | 6.152335401 | 3.07E-08 | 7.13E-07 |
| MT1F | -0.689649563 | 8.48799146 | 3.24E-08 | 7.46E-07 |
| KLRB1 | 0.736764772 | 4.328110897 | 3.69E-08 | 8.32E-07 |
| CYP27B1 | -1.316278686 | 5.390377932 | 3.79E-08 | 8.41E-07 |
| SLPI | 1.161983051 | 5.768091863 | 3.92E-08 | 8.62E-07 |
| GALNT7 | 0.604033355 | 5.039176427 | 4.12E-08 | 9.00E-07 |
| CD83 | -0.656079097 | 5.574664669 | 4.17E-08 | 9.07E-07 |
| HLA-DRA | 0.894344122 | 7.211164696 | 4.39E-08 | 9.40E-07 |
| GMFG | 0.776710111 | 4.719643598 | 4.48E-08 | 9.55E-07 |
| HCLS1 | 0.881577241 | 4.475559266 | 4.54E-08 | 9.62E-07 |
| MNS1 | 0.630975717 | 4.111293806 | 4.62E-08 | 9.74E-07 |
| ZNF331 | -0.583748756 | 4.606978316 | 4.64E-08 | 9.74E-07 |
| NPHS2 | -1.381029447 | 4.924396319 | 4.76E-08 | 9.96E-07 |
| TYROBP | 1.04128831 | 5.096378017 | 4.78E-08 | 9.98E-07 |
| RASSF2 | 0.644615049 | 3.580070273 | 4.80E-08 | 9.98E-07 |
| HPN | -0.641300445 | 6.47116152 | 5.01E-08 | 1.03E-06 |
| RNASE6 | 0.925064645 | 4.338063584 | 5.28E-08 | 1.07E-06 |
| EMP3 | 0.689336243 | 4.983912942 | 5.60E-08 | 1.12E-06 |
| ETNK2 | -0.649643057 | 4.961316434 | 6.09E-08 | 1.20E-06 |
| HLA-DPB1 | 0.925030906 | 6.228864461 | 6.20E-08 | 1.22E-06 |
| RAC2 | 0.636122023 | 4.857381132 | 6.48E-08 | 1.27E-06 |
| SLC12A3 | -0.652378202 | 5.436177022 | 6.56E-08 | 1.28E-06 |
| MS4A6A | 0.922071884 | 5.099157176 | 6.62E-08 | 1.29E-06 |
| CIDEB | -0.572252415 | 6.047590634 | 7.26E-08 | 1.40E-06 |
| CAV1 | 0.648552202 | 4.461848371 | 7.64E-08 | 1.45E-06 |
| RAB11FIP3 | -0.591649537 | 5.734670066 | 7.64E-08 | 1.45E-06 |
| FTCD | -0.895820024 | 6.151227089 | 7.73E-08 | 1.47E-06 |
| CD2 | 0.628369372 | 4.083591195 | 8.07E-08 | 1.51E-06 |
| TESC | 0.730240526 | 4.552602032 | 8.15E-08 | 1.52E-06 |
| TM4SF1 | 0.602459226 | 4.743935858 | 8.37E-08 | 1.56E-06 |
| WFDC2 | 1.006477662 | 6.25389326 | 8.44E-08 | 1.56E-06 |
| S100A4 | 0.818885288 | 5.581636916 | 8.78E-08 | 1.61E-06 |
| GLIPR1 | 0.513656221 | 4.209074471 | 9.70E-08 | 1.76E-06 |
| SERPING1 | 0.807665836 | 6.030921144 | 1.01E-07 | 1.82E-06 |
| SLC5A2 | -0.651808257 | 6.492138079 | 1.01E-07 | 1.82E-06 |
| FKBP11 | 0.611848508 | 5.314253452 | 1.07E-07 | 1.89E-06 |
| CEBPD | -0.507451189 | 7.129207458 | 1.12E-07 | 1.97E-06 |
| PPARGC1A | -0.507624466 | 5.439333984 | 1.14E-07 | 1.99E-06 |
| CPNE6 | -0.846895694 | 4.027864431 | 1.18E-07 | 2.05E-06 |
| ARL4C | 0.82994159 | 4.419480879 | 1.25E-07 | 2.16E-06 |
| C3 | 1.315117077 | 6.387366481 | 1.28E-07 | 2.20E-06 |
| PSMB9 | 0.852601557 | 4.917006842 | 1.28E-07 | 2.20E-06 |
| CST6 | 0.511914079 | 3.266397564 | 1.29E-07 | 2.21E-06 |
| CD1C | 0.515268003 | 4.752667679 | 1.31E-07 | 2.23E-06 |
| KCTD12 | 0.545435151 | 5.984443675 | 1.49E-07 | 2.50E-06 |
| PLA2G4A | 0.529610487 | 3.274654331 | 1.63E-07 | 2.70E-06 |
| EPHX1 | -0.586665657 | 6.267149633 | 1.79E-07 | 2.91E-06 |
| GCH1 | -0.676694164 | 6.372790794 | 1.85E-07 | 2.98E-06 |
| IL10RA | 0.687154154 | 4.045032299 | 1.86E-07 | 2.99E-06 |
| SALL1 | -0.554600445 | 4.951483752 | 1.86E-07 | 2.99E-06 |
| APOC3 | -0.912956488 | 4.394276449 | 1.90E-07 | 3.04E-06 |
| SRGN | 0.787203994 | 5.782344655 | 2.01E-07 | 3.17E-06 |
| MAFF | -1.046901983 | 4.453410812 | 2.11E-07 | 3.32E-06 |
| CYFIP2 | -0.513335761 | 6.440100029 | 2.13E-07 | 3.33E-06 |
| FCGR2B | 0.558856273 | 3.676203581 | 2.49E-07 | 3.80E-06 |
| TM7SF3 | -0.535859916 | 4.365268423 | 2.61E-07 | 3.93E-06 |
| CKLF | 0.606531824 | 4.456952697 | 2.64E-07 | 3.97E-06 |
| FCN1 | 0.506261028 | 3.529939344 | 2.72E-07 | 4.04E-06 |
| BASP1 | 0.789296117 | 4.997969043 | 2.72E-07 | 4.04E-06 |
| NFIL3 | -0.606631627 | 5.608760794 | 2.88E-07 | 4.22E-06 |
| CCL19 | 1.146449907 | 4.234783818 | 2.97E-07 | 4.32E-06 |
| CPVL | 0.598050997 | 6.116340139 | 3.13E-07 | 4.48E-06 |
| S100A13 | 0.564349824 | 6.54150692 | 3.13E-07 | 4.48E-06 |
| CFH | 0.829279544 | 4.341247893 | 3.39E-07 | 4.80E-06 |
| ECHDC3 | -0.616414165 | 7.23384169 | 3.41E-07 | 4.82E-06 |
| KNG1 | -0.784307275 | 6.081512053 | 3.56E-07 | 5.00E-06 |
| POSTN | 1.073551651 | 4.426182912 | 3.61E-07 | 5.05E-06 |
| CLU | 0.872941758 | 6.538005032 | 3.75E-07 | 5.21E-06 |
| CAV2 | 0.50470835 | 4.393263148 | 3.91E-07 | 5.41E-06 |
| LY75 | 0.700126093 | 3.738792781 | 3.97E-07 | 5.46E-06 |
| CA2 | -0.621074375 | 7.826273888 | 4.06E-07 | 5.57E-06 |
| LTB | 0.695864814 | 4.621427151 | 4.07E-07 | 5.59E-06 |
| CD52 | 0.922803777 | 4.362918324 | 4.26E-07 | 5.82E-06 |
| DSE | 0.569393134 | 4.835650449 | 4.30E-07 | 5.86E-06 |
| SLC34A1 | -0.924630349 | 6.039049918 | 4.31E-07 | 5.87E-06 |
| ASPA | -0.848487148 | 6.482899406 | 4.61E-07 | 6.20E-06 |
| CD163 | 0.630552625 | 4.57505893 | 4.80E-07 | 6.36E-06 |
| ALDH1B1 | -0.51008396 | 5.377021381 | 4.84E-07 | 6.40E-06 |
| SLC2A4RG | -0.510406251 | 5.299970948 | 4.92E-07 | 6.51E-06 |
| UPB1 | -0.980729841 | 5.622130511 | 4.93E-07 | 6.51E-06 |
| ACSM5 | -0.592961452 | 5.257918002 | 5.08E-07 | 6.68E-06 |
| IRF8 | 0.741070492 | 5.087348427 | 5.19E-07 | 6.78E-06 |
| ALDH1L1 | -0.543496919 | 6.737892494 | 5.27E-07 | 6.86E-06 |
| HSD11B2 | -0.527598825 | 6.683719513 | 5.71E-07 | 7.31E-06 |
| HLA-B | 0.66108605 | 8.149115098 | 5.78E-07 | 7.38E-06 |
| TRAF5 | 0.521898772 | 3.71473895 | 5.98E-07 | 7.58E-06 |
| GIMAP6 | 0.582244421 | 3.773040032 | 5.99E-07 | 7.58E-06 |
| ARPC1B | 0.751117962 | 5.950337039 | 6.02E-07 | 7.60E-06 |
| ACAA1 | -0.546721454 | 7.034119236 | 6.41E-07 | 8.03E-06 |
| SPARC | 0.719235004 | 5.752138514 | 6.61E-07 | 8.22E-06 |
| TYRP1 | -0.874997384 | 4.548886652 | 6.61E-07 | 8.22E-06 |
| GNAI1 | 0.538946678 | 4.535722768 | 6.63E-07 | 8.22E-06 |
| PMP22 | 0.556906037 | 5.029171989 | 6.87E-07 | 8.43E-06 |
| THY1 | -0.824074783 | 6.537300574 | 6.94E-07 | 8.49E-06 |
| GHR | -0.738111239 | 5.148631297 | 8.06E-07 | 9.65E-06 |
| ATF3 | -0.972963427 | 4.428118222 | 8.11E-07 | 9.69E-06 |
| DKK3 | 0.529461973 | 3.490178243 | 9.08E-07 | 1.07E-05 |
| FCER1G | 0.642222965 | 5.393529911 | 9.16E-07 | 1.08E-05 |
| SGK1 | -0.649332903 | 7.31661213 | 9.72E-07 | 1.12E-05 |
| GLUD1 | -0.508830921 | 7.126028237 | 9.76E-07 | 1.12E-05 |
| SPAG5 | -0.598095577 | 5.435392927 | 1.03E-06 | 1.18E-05 |
| PSMB8 | 0.600356439 | 5.268089848 | 1.05E-06 | 1.19E-05 |
| ALDH4A1 | -0.625387407 | 6.586531474 | 1.06E-06 | 1.20E-05 |
| CTH | -0.528813023 | 4.768242631 | 1.09E-06 | 1.23E-05 |
| TUBB6 | 0.697265184 | 4.988661504 | 1.09E-06 | 1.23E-05 |
| CXCL6 | 2.518381342 | 2.808665336 | 1.18E-06 | 1.32E-05 |
| RFTN1 | 0.604440368 | 5.302557487 | 1.21E-06 | 1.34E-05 |
| ARHGAP29 | 0.563814184 | 6.46252325 | 1.22E-06 | 1.35E-05 |
| ACADL | -0.513641327 | 5.111588322 | 1.24E-06 | 1.36E-05 |
| TNMD | 0.704053215 | 3.888651441 | 1.25E-06 | 1.37E-05 |
| MEIS2 | 0.570360961 | 4.509159314 | 1.33E-06 | 1.45E-05 |
| PCSK1N | -0.597076904 | 5.896150856 | 1.34E-06 | 1.46E-05 |
| CXCL1 | 0.60496007 | 3.560054672 | 1.47E-06 | 1.58E-05 |
| CLIC5 | -0.705948254 | 4.184353291 | 1.53E-06 | 1.64E-05 |
| ASNS | 0.500563502 | 3.755632255 | 1.55E-06 | 1.65E-05 |
| C1QB | 0.919756517 | 4.612847711 | 1.59E-06 | 1.69E-05 |
| LPL | -0.709218206 | 4.016077228 | 1.67E-06 | 1.75E-05 |
| DUSP9 | -0.505699194 | 4.531964447 | 1.71E-06 | 1.79E-05 |
| EGR1 | -0.81460236 | 5.044189339 | 1.83E-06 | 1.87E-05 |
| HLA-F | 0.513335452 | 6.955015457 | 1.88E-06 | 1.91E-05 |
| CD3D | 0.662918847 | 4.080462665 | 1.89E-06 | 1.92E-05 |
| PPP1R16B | -0.647984551 | 5.825167695 | 1.96E-06 | 1.96E-05 |
| S100A11 | 0.605512627 | 6.573251447 | 2.07E-06 | 2.05E-05 |
| CAPG | 0.534769083 | 5.821586878 | 2.16E-06 | 2.11E-05 |
| RRAS | 0.502738826 | 4.443433275 | 2.19E-06 | 2.13E-05 |
| AKAP12 | 0.657386244 | 5.999198004 | 2.20E-06 | 2.14E-05 |
| SELL | 0.688457671 | 3.730371683 | 2.21E-06 | 2.14E-05 |
| IFI27 | 0.784830829 | 5.717059829 | 2.26E-06 | 2.19E-05 |
| SGCE | 0.502597 | 5.628069372 | 2.49E-06 | 2.38E-05 |
| CCL5 | 0.642823525 | 3.708114621 | 2.73E-06 | 2.58E-05 |
| PRODH2 | -0.783461655 | 7.601206489 | 2.75E-06 | 2.59E-05 |
| HLA-DMB | 0.609670009 | 5.887834617 | 2.85E-06 | 2.66E-05 |
| IER5 | 0.61459321 | 4.000901818 | 3.03E-06 | 2.80E-05 |
| TIPARP | -0.62242148 | 5.832176673 | 3.06E-06 | 2.81E-05 |
| GZMK | 0.64921571 | 4.492987092 | 3.39E-06 | 3.06E-05 |
| MTHFD2 | 0.752208469 | 3.811103543 | 3.48E-06 | 3.14E-05 |
| KLHL3 | -0.550643914 | 5.322169675 | 3.66E-06 | 3.25E-05 |
| DPP4 | -0.587395009 | 6.515471193 | 3.79E-06 | 3.32E-05 |
| RNF186 | -0.639503763 | 6.279770954 | 4.12E-06 | 3.57E-05 |
| SLC7A7 | -0.70278589 | 8.217394675 | 4.19E-06 | 3.62E-05 |
| S100A6 | 0.570290973 | 7.068768174 | 4.41E-06 | 3.78E-05 |
| VCAM1 | 0.631357701 | 7.634480202 | 4.43E-06 | 3.79E-05 |
| ISG20 | 0.5619475 | 3.788655963 | 4.65E-06 | 3.92E-05 |
| QDPR | -0.501875555 | 7.79027917 | 4.74E-06 | 3.96E-05 |
| GPC3 | -0.596371903 | 5.27136039 | 4.89E-06 | 4.04E-05 |
| FOS | -1.172645722 | 4.707516801 | 5.11E-06 | 4.17E-05 |
| CYP4A11 | -0.666121506 | 5.699282414 | 5.19E-06 | 4.22E-05 |
| SRPX | 0.546974973 | 3.522324094 | 5.67E-06 | 4.56E-05 |
| C1R | 0.781280989 | 5.44561552 | 5.90E-06 | 4.72E-05 |
| MGP | 0.839158112 | 7.206958345 | 6.03E-06 | 4.80E-05 |
| VSIG4 | 0.593667803 | 4.642115954 | 6.17E-06 | 4.91E-05 |
| AFM | -1.131874352 | 5.505451542 | 6.23E-06 | 4.93E-05 |
| HPGD | -0.683496499 | 5.492045089 | 6.99E-06 | 5.42E-05 |
| SERPINE2 | 0.543382054 | 7.038006057 | 7.20E-06 | 5.55E-05 |
| C1QA | 0.779399594 | 4.768469338 | 7.41E-06 | 5.69E-05 |
| JUNB | -0.540826119 | 4.664783575 | 8.07E-06 | 6.13E-05 |
| ANK2 | -0.679184128 | 6.253240765 | 8.52E-06 | 6.42E-05 |
| TSPAN8 | 0.783240945 | 5.787772233 | 9.96E-06 | 7.33E-05 |
| ID1 | 0.635616259 | 6.056158283 | 1.04E-05 | 7.59E-05 |
| SERPINA6 | -0.737633522 | 6.043768073 | 1.07E-05 | 7.77E-05 |
| SGMS1 | 0.501147802 | 4.364893267 | 1.10E-05 | 7.95E-05 |
| APOH | -1.150349388 | 5.650091048 | 1.13E-05 | 8.13E-05 |
| HLA-DMA | 0.624947428 | 5.975553795 | 1.19E-05 | 8.50E-05 |
| SMPDL3A | -0.641210548 | 6.52272199 | 1.24E-05 | 8.75E-05 |
| SOX4 | 0.566669668 | 5.454414451 | 1.43E-05 | 9.87E-05 |
| ACADSB | -0.576719662 | 5.797329278 | 1.49E-05 | 0.000102328 |
| ARSF | -0.519288689 | 5.620367878 | 1.68E-05 | 0.000112348 |
| KCNK5 | -0.524066536 | 5.738997414 | 1.71E-05 | 0.000113467 |
| ACOX2 | -0.621137476 | 6.383057091 | 1.74E-05 | 0.000115096 |
| BLNK | 0.506154154 | 5.430861947 | 1.87E-05 | 0.000122785 |
| LCN2 | 0.804641502 | 4.208666402 | 1.88E-05 | 0.000123106 |
| ENTPD5 | -0.527567474 | 5.827408543 | 1.95E-05 | 0.000127218 |
| TFRC | -0.517265275 | 5.635197658 | 1.96E-05 | 0.000127477 |
| CXCL12 | 0.508436039 | 6.301651615 | 2.47E-05 | 0.000154791 |
| SERPINF2 | -0.544537121 | 5.220555141 | 2.86E-05 | 0.000174836 |
| IGHM | 0.813329123 | 4.736945809 | 2.97E-05 | 0.000180207 |
| RHOB | -0.502108666 | 6.650684538 | 3.00E-05 | 0.000182029 |
| CLC | 0.569591048 | 3.747743961 | 3.15E-05 | 0.000190127 |
| FGF9 | -0.507236785 | 4.964393928 | 3.22E-05 | 0.000193567 |
| GSTA1 | -0.69750877 | 8.268151159 | 3.38E-05 | 0.00020182 |
| SLC22A4 | -0.578615989 | 5.378303717 | 3.88E-05 | 0.000225837 |
| IGFBP6 | 0.618962324 | 5.312108503 | 4.10E-05 | 0.000236745 |
| FKBP5 | -0.598697 | 5.183400795 | 4.24E-05 | 0.000243956 |
| CLEC10A | 0.525786875 | 3.675461222 | 4.28E-05 | 0.000245317 |
| ALB | -1.301872898 | 6.352642601 | 5.12E-05 | 0.000284757 |
| KL | -0.674795086 | 7.175383624 | 5.18E-05 | 0.000287873 |
| HBB | 1.262793424 | 6.591252292 | 5.26E-05 | 0.000291578 |
| ACSF2 | -0.520681971 | 6.606976593 | 5.50E-05 | 0.000302762 |
| MME | -0.692485545 | 7.110102405 | 6.44E-05 | 0.000344631 |
| BHMT2 | -0.535113348 | 8.018038609 | 6.63E-05 | 0.000352098 |
| ADH1B | 0.757252796 | 5.679937 | 7.28E-05 | 0.000379424 |
| SERPINF1 | 0.690471418 | 5.334126596 | 7.29E-05 | 0.000379646 |
| FBP1 | -0.670612277 | 7.780133963 | 7.39E-05 | 0.000383353 |
| SLC22A8 | -0.808620493 | 6.984570797 | 8.68E-05 | 0.000437582 |
| AGXT | -0.655774603 | 5.089886046 | 8.92E-05 | 0.000447689 |
| SLC13A3 | -0.575336198 | 7.013475062 | 9.71E-05 | 0.000481106 |
| FMO3 | 0.528052873 | 3.921897995 | 9.89E-05 | 0.000489408 |
| GJA1 | 0.749976187 | 4.877603688 | 0.000109102 | 0.000527848 |
| RARRES1 | 0.760637153 | 4.367237724 | 0.000113443 | 0.000542309 |
| DPYS | -0.679022739 | 7.279614823 | 0.00012577 | 0.000589117 |
| PFKFB3 | 0.598219292 | 5.901849505 | 0.000126886 | 0.000593164 |
| C7 | 0.629028372 | 6.915437092 | 0.000138409 | 0.000638471 |
| HMGCS2 | -0.576627892 | 5.459078587 | 0.000139339 | 0.000642447 |
| DAO | -0.627945814 | 6.366178729 | 0.000149043 | 0.000679207 |
| FGF1 | -0.586465407 | 3.996813462 | 0.000152802 | 0.000693318 |
| SERPINA3 | 0.996976218 | 5.985271424 | 0.000153572 | 0.000695863 |
| XPNPEP2 | -0.673015777 | 6.533576931 | 0.000156119 | 0.000705985 |
| JUN | -0.517640394 | 4.754618957 | 0.000163822 | 0.00073377 |
| PTPRO | -0.607598724 | 3.722974345 | 0.000174198 | 0.000773987 |
| GBP2 | 0.507077221 | 3.820084989 | 0.000179641 | 0.000793301 |
| DPEP1 | -0.752797132 | 7.086167527 | 0.000193066 | 0.000841924 |
| SLC10A2 | -0.596781818 | 5.605208458 | 0.000199493 | 0.000865985 |
| SLC22A11 | -0.511533031 | 6.109756396 | 0.000210495 | 0.00090784 |
| TREH | -0.658668738 | 4.922206772 | 0.0002237 | 0.000953865 |
| KCNN2 | -0.693015431 | 3.878543487 | 0.000230788 | 0.000976896 |
| PLCL1 | -0.526755669 | 5.174559053 | 0.000232588 | 0.000983301 |
| PCOLCE2 | -0.80939998 | 3.062827044 | 0.000261161 | 0.001074196 |
| PLTP | 0.580371597 | 5.168674681 | 0.000272307 | 0.001112331 |
| SLC22A6 | -0.585206532 | 6.662984525 | 0.000285195 | 0.001154392 |
| ISG15 | 0.538309731 | 5.084172249 | 0.000288805 | 0.001167498 |
| ALDH6A1 | -0.514392549 | 7.767470815 | 0.00029125 | 0.001175361 |
| PSMB10 | 0.539613625 | 4.635039596 | 0.000297641 | 0.001197558 |
| BST2 | 0.55775997 | 5.090425981 | 0.000328015 | 0.001298134 |
| CXCL9 | 0.546957839 | 4.14910625 | 0.000361341 | 0.001403545 |
| GDF15 | -0.650982189 | 5.698389876 | 0.000383514 | 0.001470786 |
| SPINK1 | -0.777064622 | 5.513571205 | 0.000387297 | 0.001483484 |
| SFN | 0.523413733 | 5.247651462 | 0.00039359 | 0.001501636 |
| MT1M | -0.663430585 | 4.913645262 | 0.000422817 | 0.001594242 |
| GLYAT | -0.555498575 | 7.25196496 | 0.000477811 | 0.00176139 |
| AKR7A3 | -0.512946927 | 6.984750501 | 0.000527915 | 0.001914606 |
| SLC2A2 | -0.584268261 | 5.574377317 | 0.000607443 | 0.002159772 |
| PCK1 | -0.514791544 | 8.347773412 | 0.000612183 | 0.002171705 |
| AZGP1 | -0.662295014 | 6.944297398 | 0.000650839 | 0.002289852 |
| BHMT | -0.610542401 | 8.732494972 | 0.000696483 | 0.002417023 |
| CHI3L1 | -0.604817004 | 3.49488015 | 0.000903076 | 0.003006527 |
| HLA-DQA1 | 0.842143128 | 4.716346081 | 0.001030196 | 0.003363141 |
| DCN | 0.564949918 | 6.492022499 | 0.001166832 | 0.003745557 |
| ANGPTL3 | -0.686293397 | 5.769321525 | 0.001243085 | 0.003959286 |
| HAO2 | -0.630295712 | 7.102897933 | 0.001287954 | 0.004078394 |
| DCXR | -0.534816517 | 7.990454513 | 0.001300881 | 0.004112758 |
| KMO | -0.569511579 | 6.296253658 | 0.001328419 | 0.004182963 |
| MAFB | 0.554152697 | 4.962535652 | 0.001353134 | 0.004240931 |
| MNDA | 1.398917457 | 2.30958576 | 0.001475246 | 0.00455541 |
| GPNMB | 0.529725731 | 5.300537285 | 0.00156906 | 0.004784555 |
| CR2 | -0.700019586 | 4.17863363 | 0.002047407 | 0.005995749 |
| CALB1 | -0.547144044 | 7.750811905 | 0.002260817 | 0.006499418 |
| DIO1 | -0.563804398 | 7.220255729 | 0.002261711 | 0.006500003 |
| HLA-DQB1 | 0.617539885 | 5.053863136 | 0.00277075 | 0.007697436 |
| SLC7A9 | -0.52122078 | 7.556335708 | 0.00395101 | 0.01035024 |
| GPC5 | -0.588874698 | 4.225275115 | 0.004434741 | 0.011423552 |
| ZGPAT | -0.514246364 | 5.963938368 | 0.005767623 | 0.014240914 |
| CD69 | 1.071438659 | 2.043875772 | 0.005846334 | 0.014412996 |
| SAMSN1 | 1.13029483 | 2.141266635 | 0.006254142 | 0.015260033 |
| ZEB2 | 1.081597033 | 2.021092991 | 0.006993912 | 0.016750873 |
| UMOD | -0.636818258 | 8.672299793 | 0.007539996 | 0.017867874 |
| HPD | -0.619287935 | 7.77504036 | 0.007544665 | 0.017872597 |
| PODXL | -0.578930719 | 5.860257847 | 0.007969506 | 0.018753536 |
| APOBEC3B | 1.04143028 | 2.152955595 | 0.008326011 | 0.019459339 |
| FABP1 | -0.524848641 | 7.582807566 | 0.008327316 | 0.019459339 |
| NFE2L3 | 1.034863608 | 1.94494163 | 0.008602663 | 0.020008417 |
| SACS | 1.010402511 | 2.09705669 | 0.009779754 | 0.022348638 |
| ECT2 | 0.994177429 | 2.029157879 | 0.010489143 | 0.023727842 |
| PLG | -0.596270643 | 7.29671018 | 0.012956314 | 0.028466343 |
| FCGR3B | 1.094229529 | 2.268677081 | 0.014800921 | 0.031849994 |
| VNN1 | -0.524106917 | 5.187265822 | 0.018417923 | 0.038235749 |
| GZMB | 0.947480076 | 2.141554148 | 0.018421387 | 0.038235749 |
| CLEC4A | 0.942138092 | 2.140380734 | 0.018713175 | 0.038790079 |
| S100A8 | 0.607927003 | 4.730521927 | 0.019140711 | 0.039511026 |
| REG1A | 0.543325609 | 4.385495605 | 0.019633124 | 0.040385813 |
| GPR65 | 0.914904654 | 2.162250643 | 0.019659286 | 0.040421964 |
| ADCY7 | 0.935304313 | 2.08570693 | 0.020765453 | 0.042344972 |
| HHEX | 0.878095318 | 2.035317304 | 0.024185577 | 0.047928268 |
