# Supplementary material for: Identification of immune-associated biomarkers of diabetes nephropathy tubulointerstitial injury based on machine learning: a bioinformatics multi-chip integrated analysis
Source: BioData Min. 2024 Jul 1;17:20. doi: 10.1186/s13040-024-00369-x (PMC11218417; doi:10.1186/s13040-024-00369-x)
Supplement: Supplementary file 7 — Supplementary Material 7 [file 13040_2024_369_MOESM7_ESM.docx]

**Supplementary TABLE 6:** SVM-RFE algorithm screening characteristic genes for DN.

|  | FeatureName | AvgRank |
| --- | --- | --- |
| 1 | REG1A | 6 |
| 2 | DEFB1 | 8 |
| 3 | AGR2 | 8.2 |
| 4 | CISH | 9.3 |
| 5 | S100A8 | 10.4 |
| 6 | PLTP | 13.8 |
| 7 | CCR2 | 14.8 |
| 8 | PLSCR1 | 15.1 |
| 9 | CLEC10A | 15.5 |
| 10 | CEBPD | 15.7 |
| 11 | FSTL1 | 16.1 |
| 12 | JUN | 17.1 |
| 13 | PTGER3 | 17.4 |
| 14 | CX3CR1 | 20 |
| 15 | CD53 | 20.2 |
| 16 | ITGAM | 20.4 |
