# Supplementary figures and images for: Identification of immune-associated biomarkers of diabetes nephropathy tubulointerstitial injury based on machine learning: a bioinformatics multi-chip integrated analysis
Source: BioData Min. 2024 Jul 1;17:20. doi: 10.1186/s13040-024-00369-x (PMC11218417; doi:10.1186/s13040-024-00369-x)

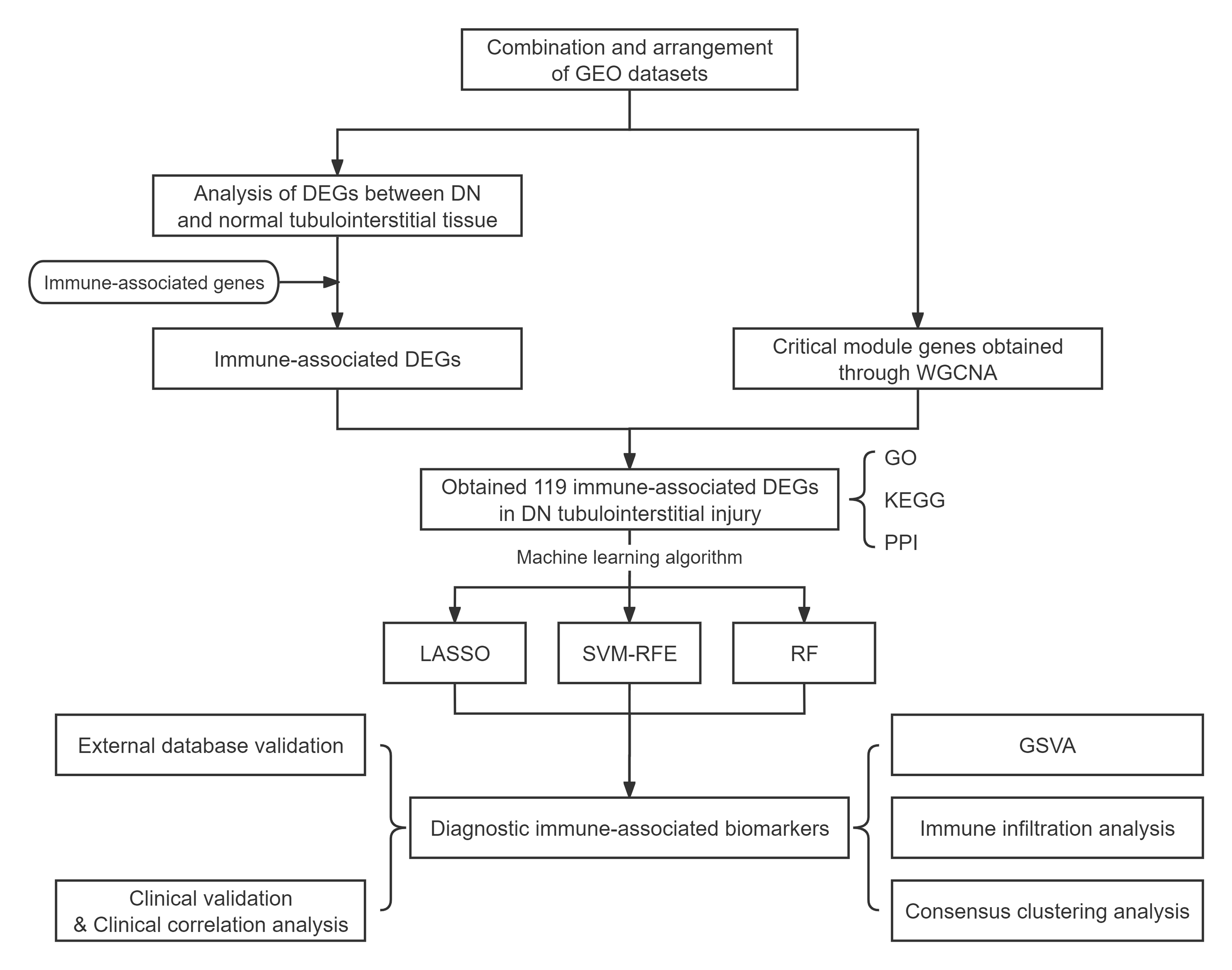

Supplement: Supplementary file 8 — Supplementary Material 8 [file 13040_2024_369_MOESM8_ESM.tif]
